# Supplementary material for: Clinical factors associated with progression to dementia in people with late-life depression: a cohort study of patients in secondary care
Source: BMJ Open. 2020 May 24;10(5):e035147. doi: 10.1136/bmjopen-2019-035147 (PMC7252968; doi:10.1136/bmjopen-2019-035147)
Supplement: Supplementary data [file bmjopen-2019-035147supp001.pdf]

Supplementary Table 1: Multivariate Cox proportional hazards models analysing associations between predictor variables and risk of dementia diagnosis (adjusted hazard ratio (95% CI)) in the complete case sample of 2,952 late-life depression patients, of whom 717 (24.3%) were diagnosed with dementia during the study follow-up.

Predictor variables	**Model 1** Adjusted for age, gender, deprivation	**Model 2** Model 1 + depressed mood[b] + cognitive problems[b]	**Model 3** Adjusted for all predictor variables
**Demographics[a]**			
Centred age (per year difference from mean)	1.07 (1.06-1.08)**	1.05 (1.03-1.06)**	1.04 (1.03-1.06)**
*Centred age * time[c]*		*1.01 (1.00-1.01)**	*1.01 (1.00-1.01)**
Female gender	0.88 (0.75-1.03)	0.87 (0.75-1.03)	0.90 (0.76-1.06)
Non-white ethnicity	1.33 (1.10-1.61)*	1.21 (1.00-1.46)*	1.14 (0.94-1.39)
Married or cohabiting status[a]	1.04 (0.88-1.23)	1.08 (0.91-1.27)	1.07 (0.90-1.27)
Deprivation (per 10 unit increase in IMD score)	1.01 (0.95-1.08)	0.99 (0.93-1.06)	1.00 (0.94-1.06)
Recurrent depressive disorder diagnosis or previous diagnosis of depression	0.53 (0.39-0.70)**	0.71 (0.60-0.84)**	0.72 (0.60-0.86)**
*Recurrent depression * time[c]*	*1.08 (1.01-1.15)**		
**HoNOS65+ mental and physical health problems[a,b]**			
Agitated behaviour	1.06 (0.87-1.30)	0.88 (0.72-1.08)	0.97 (0.78-1.21)
Non-accidental self-injury	0.70 (0.54-0.90)*	0.69 (0.53-0.91)*	0.77 (0.59-1.01)
Drug/alcohol problems	1.04 (0.72-1.50)	0.98 (0.68-1.41)	1.08 (0.76-1.53)
Hallucinations and delusions	0.84 (0.67-1.07)	0.53 (0.36-0.78)**	0.52 (0.35-0.78)*
*Hallucinations and delusion * time[c]*		*1.10 (1.00-1.21)**	*1.13 (1.02-1.25)**
Depressed mood	1.09 (0.94-1.27)	1.00 (0.86-1.16)	1.07 (0.90-1.26)
Physical illness or disability	1.27 (1.08-1.48)*	1.14 (0.98-1.34)	0.90 (0.70-1.16)
*Physical illness or disability * time[c]*			*1.06 (0.99-1.13)*
Cognitive problems	4.84 (3.75-6.24)**	4.91 (3.80-6.34)**	4.66 (3.58-6.08)**
*Cognitive problems * time[c]*	*0.83 (0.77-0.90)***	*0.83 (0.77-0.90)***	*0.83 (0.76-0.90)***
**HoNOS65+ functional problems[a,b]**			
Activities of daily living	1.38 (1.18-1.60)**	1.10 (0.93-1.29)	1.04 (0.87-1.24)
Living conditions	1.05 (0.83-1.31)	0.90 (0.71-1.15)	0.90 (0.71-1.16)
Occupational/recreational activities	1.11 (0.95-1.29)	1.02 (0.86-1.20)	1.06 (0.89-1.27)
Social relationships	1.09 (0.83-1.42)	1.04 (0.79-1.36)	1.14 (0.86-1.51)
*Social relationships * time[c]*	*0.92 (0.85-0.99)**	*0.92 (0.86-1.00)**	*0.91 (0.84-0.98)**
**Psychotropic use: in 2 years pre-depression diagnosis**			
Antidepressant	0.86 (0.74-1.00)*	0.88 (0.76-1.03)	0.91 (0.78-1.06)
**Psychotropic use: post-depression diagnosis**			
Any antidepressant	1.12 (0.86-1.46)	1.17 (0.90-1.52)	1.30 (0.97-1.73)
SSRI	1.03 (0.88-1.19)	1.00 (0.86-1.17)	0.98 (0.83-1.15)
Antipsychotic	0.86 (0.74-1.00)*	0.85 (0.73-0.99)*	0.98 (0.83-1.16)
**Cognitive behavioural therapy**			
Received CBT post-depression diagnosis	0.59 (0.50-0.70)**	0.66 (0.55-0.79)**	0.70 (0.58-0.85)**

*p<.05
**p<.002 (Bonferroni corrected)
[a] at or closest to the time of depression diagnosis
[b] Health of the Nation Outcome Scale 65+ (HoNOS65+) subscale scores 0-4 (0 = least severe, 4 = most severe status). Values represent frequencies of patients scored as experiencing problems (score 2-4) in that domain.
[c] time-variable-interactions describing how the hazard in the variable reported above changes per year.
