# Supplementary material for: Clinical factors associated with progression to dementia in people with late-life depression: a cohort study of patients in secondary care
Source: BMJ Open. 2020 May 24;10(5):e035147. doi: 10.1136/bmjopen-2019-035147 (PMC7252968; doi:10.1136/bmjopen-2019-035147)
Supplement: Author's manuscript [file bmjopen-2019-035147.draft_revisions.pdf]

BMJ Open is committed to open peer review. As part of this commitment we make the peer review history of every article we publish publicly available.

When an article is published we post the peer reviewers' comments and the authors' responses online. We also post the versions of the paper that were used during peer review. These are the versions that the peer review comments apply to.

The versions of the paper that follow are the versions that were submitted during the peer review process. They are not the versions of record or the final published versions. They should not be cited or distributed as the published version of this manuscript.

BMJ Open is an open access journal and the full, final, typeset and author-corrected version of record of the manuscript is available on our site with no access controls, subscription charges or pay-per-view fees (http://bmjopen.bmj.com).

If you have any questions on BMJ Open's open peer review process please email info.bmjopen@bmj.com

BMJ Open

Clinical factors associated with progression to dementia in people with late-life depression: a cohort study of patients in secondary care

Journal:	*BMJ Open*
Manuscript ID	bmjopen-2019-035147
Article Type:	Original research
Date Submitted by the Author:	21-Oct-2019

Complete List of Authors:	Peakman, Georgia; IoPPN, Old Age Psychiatry;<br>Karunatilake, Nishshanka; South London and Maudsley NHS Foundation Trust<br>Seynaeve, Mathieu; South London and Maudsley NHS Foundation Trust; King's College London Institute of Psychiatry<br>Perera, Gayan; Institute of Psychiatry, Psychological Medicine<br>Aarsland, Dag; Stavanger University Hospital; King's College London Institute of Psychiatry, Department of Old Age Psychiatry<br>Stewart, Robert; King's College London, Institute of Psychiatry; South London and Maudsley NHS Foundation Trust<br>Mueller, Christoph; King's College London Institute of Psychiatry, Department of Old Age Psychiatry; South London and Maudsley NHS Foundation Trust

Keywords:	Depression & mood disorders < PSYCHIATRY, Dementia < NEUROLOGY, GERIATRIC MEDICINE, MENTAL HEALTH

SCHOLARONE™
Manuscripts

I, the Submitting Author has the right to grant and does grant on behalf of all authors of the Work (as defined in the below author licence), an exclusive licence and/or a non-exclusive licence for contributions from authors who are: i) UK Crown employees; ii) where BMJ has agreed a CC-BY licence shall apply, and/or iii) in accordance with the terms applicable for US Federal Government officers or employees acting as part of their official duties; on a worldwide, perpetual, irrevocable, royalty-free basis to BMJ Publishing Group Ltd ("BMJ") its licensees and where the relevant Journal is co-owned by BMJ to the co-owners of the Journal, to publish the Work in this journal and any other BMJ products and to exploit all rights, as set out in our licence.

The Submitting Author accepts and understands that any supply made under these terms is made by BMJ to the Submitting Author unless you are acting as an employee on behalf of your employer or a postgraduate student of an affiliated institution which is paying any applicable article publishing charge ("APC") for Open Access articles. Where the Submitting Author wishes to make the Work available on an Open Access basis (and intends to pay the relevant APC), the terms of reuse of such Open Access shall be governed by a Creative Commons licence – details of these licences and which Creative Commons licence will apply to this Work are set out in our licence referred to above.

Other than as permitted in any relevant BMJ Author's Self Archiving Policies, I confirm this Work has not been accepted for publication elsewhere, is not being considered for publication elsewhere and does not duplicate material already published. I confirm all authors consent to publication of this Work and authorise the granting of this licence.

For peer review only

Clinical factors associated with progression to dementia in people with late-life depression: a cohort study of patients in secondary care

Georgia Peakman[1], Nishshanka Karunatilake[2], Mathieu Seynaeve[1,2], Gayan Perera[1], Dag Aarsland[1,3], Robert Stewart[1,2], Christoph Mueller[1,2]

[1]King's College London, Institute of Psychiatry, Psychology and Neuroscience, London, UK

[2]South London and Maudsley NHS Foundation Trust, London, UK

[3]Centre for Age-Related Medicine, Stavanger University Hospital, Stavanger, Norway

Corresponding author: Georgia Peakman; King's College London, Institute of Psychiatry, Psychology and Neuroscience (IoPPN), De Crespigny Park, London, SE5 8AF, United Kingdom; email: georgia.peakman.18@ucl.ac.uk; phone: +44 207 848 0626.

Declarations of interest:

RS has received research funding from Roche, Pfizer, Janssen, Lundbeck and In-Silico-Bioscience. DA has received research support and/or honoraria from Astra-Zeneca, H. Lundbeck, Novartis Pharmaceuticals and GE Health, and serves as paid consultant for H. Lundbeck and Axovant. All other authors declare no conflict of interest.

Funding:

GeP received undergraduate bursaries from the Alzheimer's Society and the Wellcome Trust to conduct this project. CM, GaP, DA and RS receive salary support from the National Institute for Health Research (NIHR) Biomedical Research Centre at South London and Maudsley NHS Foundation Trust and King's College London, and RS is a NIHR Senior Investigator. The views expressed are those of the author(s) and not necessarily those of the NHS, the NIHR or the Department of Health.

For peer review only

Word count: 2960

Tables: 3

Figures: 3

For peer review only

Clinical factors associated with progression to dementia in people with late-life depression: a cohort study of patients in secondary care.

Abstract

Objectives: Depression in later life is increasingly being recognised as a prodrome for the development of dementia. We aimed to investigate which clinical factors are associated with more rapid conversion from depression to dementia.

Design: Retrospective cohort study.

Setting: South London and Maudsley NHS Foundation Trust (SLaM) secondary mental healthcare services.

Participants: The SLaM Clinical Record Interactive Search (CRIS) was used to retrieve anonymised data on 806 patients who were aged 65 years or older and received a diagnosis of depression in mental health services, followed by a diagnosis of dementia >3 months later.

Outcome measures: Predictors of accelerated dementia development were investigated including demographic factors, health status rated on the Health of the National Outcome scale for older people (HoNOS65+), depression recurrence, and treatments including psychotropic drugs and cognitive behavioural therapy (CBT).

Results: In patients with late-life depression who later received a diagnosis of dementia, significant predictors of shorter time to dementia diagnosis in fully adjusted models were older age (HR=1.03, 95% CI=1.02-1.04 per year increase), male gender (HR=0.76, 95% CI=0.64-0.89 for females), higher deprivation (HR=1.08, 95% CI=1.02-1.16 per 10 unit increase in IMD score), and HoNOS65+ subscales measuring depressed mood (HR=1.31, 95% CI=1.11-1.54 for scores of 2-4) and cognitive problems (HR=1.65, 95% CI=1.39-1.95 for scores of 2-4). Recurrent depressive disorder or past depression were protective (HR=0.79, 95% CI=0.65-0.95); associated with a longer time to dementia diagnosis. Antipsychotic drugs (HR=0.77, 95% CI=0.66-0.90) and CBT (HR=0.74, 95% CI=0.62-0.89) were significantly associated with longer times to dementia diagnosis when adjusted for demographic factors and severity of mood and cognitive difficulties.

Conclusions: In older adults with depression and subsequent dementia diagnosed by mental health services, faster progression to a diagnosis of dementia was

For peer review only

predicted by age and gender, new onset depression, and severity of depressive and cognitive symptoms.

Key words: depression, dementia, diagnosis, predictors, secondary care

For peer review only

Article Summary

Strengths and limitations of this study

- This study used a large naturalistic sample of older patients receiving routine mental healthcare, with unique access to anonymised clinical data.
- Patient follow-up was relatively long, with a mean follow-up time of 2.8 years.
- The sample was limited to patients who had received diagnoses of depression and dementia from the same provider, and the secondary care cohort is not readily generalisable to cases of depression treated in primary care.
- The influences of other comorbid conditions, risk factors such as vascular health, and resultant polypharmacy on progression to dementia were not considered.

For peer review only

Introduction

A link between depression and dementia is well established, and growing evidence indicates that the timing of depression is important in defining the nature of the relationship(1). Late-life depression has been consistently associated with up to a two-fold increased risk for dementia(2-4), and it is now recognised as a key potentially modifiable risk factor for the condition(5). However, causality is yet to be established; it remains unclear whether, and in which cases, late-life depression is an independent risk factor for dementia or a prodromal symptom.

Identification of the clinical characteristics of older adults with depression that are indicative of incipient dementia would facilitate accurate classification of individuals at high risk for early progression and subsequently, timelier treatment. The pattern and severity of depressive symptoms in late life has emerged as one important factor in determining the likelihood of progression to dementia. In two large, longitudinal cohort studies of older adults, those subjects characterised by trajectories of high and steadily increasing depressive symptoms over several years were significantly more likely to develop dementia(6, 7). Having an episode of high depressive symptoms that remitted was not associated with greater dementia risk(7). Similarly, recent data from the Whitehall II cohort study characterised those with late-life depression who later developed dementia as having a distinguishable trajectory of increasing depressive symptoms, emerging 11 years before dementia diagnosis(8). Depressive symptoms during mid-life, even when recurrent, did not increase dementia risk in this cohort. These findings, in addition to other reports of graded associations with depression severity and raised dementia risk being contingent on the two being in closer temporal proximity(9, 10), lend support to the hypotheses that depressive symptoms during later life can be an early manifestation of dementia(11), or that the two conditions share common aetiological factors(12,13); potentially both are consequences of an underlying neurodegenerative process(14).

While it is well established that greater severity of depressive symptoms is closely associated with accelerated development of dementia, other associations relevant to this relationship are less clear. Using naturalistic data from a cohort of secondary

For peer review only

mental health service users, the present study examines the associations of patient characteristics and interventions with the risk of accelerated progression from late-life depression to dementia diagnosis.

For peer review only

Methods

Source of data

Data for this study were obtained via the South London and Maudsley NHS Foundation Trust (SLaM), one of Europe's largest mental healthcare providers, serving 1.3 million residents in four south London boroughs (Croydon, Lambeth, Lewisham and Southwark), as well as delivering specialist national services. SLaM adopted fully electronic records across all of its services from 2006, having imported legacy systems which include a records system run for several years previously in dementia care. The Clinical Record Interactive Search (CRIS) application was developed in 2008 to provide access to de-identified structured and open-text data extracted from this electronic health records system, containing records on over 300,000 SLaM mental health service users(15, 16). CRIS has been enhanced by a range of natural language processing ('text-mining') applications(17) which extract structured data from text fields, including applications to ascertain pharmacotherapy(16). CRIS has research ethical approval as an anonymised database for secondary analysis (Oxford REC C 08/H0606/71+5).

Sample

Using CRIS, people aged 65 or older who received a diagnosis of depression from SLaM services within the 11-year period between 01/01/06 to 30/06/17 were identified. Cases of depression according to International Statistical Classification of Diseases and Related Health Problems 10th Revision (ICD-10)(18) codes were ascertained, both from diagnoses recorded in structured fields and in free text within clinical correspondence, through a natural language processing algorithm(17). Dementia diagnoses recorded during the study period in this late-life depression sample were obtained in the same way.

Patient and public involvement

A patient-led committee provides operational oversight of the CRIS resource and ensures that the proposed study has both scientific value as well as benefit for patients and carers (16, 19). The authors had applied to the oversight committee with an outline of the proposed analysis, including data linkages, and the project was

For peer review only - http://bmjopen.bmj.com/site/about/guidelines.xhtml

approved on 31/05/17. There was no further patient and public involvement in this register-based study.

Measurements

Late-life depression

Initial depression diagnoses (the index contact) were determined by the following diagnostic codes from ICD-10(18) within a primary or secondary diagnosis: F32 (depressive episode), F32.2 (severe depressive episode without psychotic symptoms), F32.3 (severe depressive episode with psychotic symptoms), F33 (recurrent depressive disorder), F33.2 (recurrent depressive disorder, current episode severe without psychotic symptoms), F33.3 (recurrent depressive disorder, current episode severe with psychotic symptoms). Data were also obtained for any depression diagnoses (F32, F33) recorded before the index date.

Dementia

Dementia diagnoses were determined by the following diagnostic codes from ICD-10(18) within a primary or secondary diagnosis: F00 (dementia in Alzheimer's disease), F01 (vascular dementia), F02 (dementia in other diseases classified elsewhere), F03 (unspecified dementia).

Covariates

Data were also extracted on age (at depression diagnosis), gender, ethnicity, marital or cohabiting status, local authority, a neighbourhood-level deprivation score (Index of Multiple Deprivation, 2010)(20), psychotropic medication (classified into antipsychotic, antidepressant and selective serotonin reuptake inhibitor (SSRI) types) prescribed in the period 2 years prior to depression diagnosis and following diagnosis, receipt of cognitive behaviour therapy (CBT) after depression diagnosis, and date of death up to 30/06/17. Health of the Nation Outcome Scales for older people(21) (HoNOS65+) is routinely administered in SLaM services, and the subscale scores available closest to the depression diagnosis (within 3 months of the index date) extracted for this analysis were: agitated behaviour, non-accidental self-injury, drug/alcohol problems, hallucinations or delusions, depressed mood, physical illness or disability, cognitive problems, activities of daily living (ADLs), living conditions, occupational and recreational activities, social relationships. HoNOS65+ items are rated from 0 (least

For peer review only

severe status) to 4 (most severe status). For the purpose of this analysis scores of 0-1 were considered to indicate that the patient was not experiencing problems in the domain, and scores of 2-4 were considered to indicate experiencing severe problems as rated by the clinician.

Statistical Analysis

Baseline characteristics of the sample grouped according to dementia development (diagnosed with dementia >3 months after depression diagnosis; no diagnosis of dementia in study period), were compared using chi-square tests for categorical variables, and independent samples *t*-tests or analysis of variance (ANOVA) for continuous variables.

Patients diagnosed with dementia by SLaM at least 3 months after a diagnosis of depression were included for final analysis. Cox proportional hazards regression analyses were used to investigate factors associated with time to dementia diagnosis, with the following covariates at the time of depression diagnosis: age, gender, ethnicity, marital status, deprivation, recurrent depression, psychotropic treatment, CBT treatment, and HoNOS65+ subscale scores. Time from depression diagnosis to dementia diagnosis was used as the time scale to estimate hazard ratios (HR) and 95% confidence intervals (CI). Multivariate models were first adjusted for demographic factors; age, gender and deprivation (Model 1), then for the HoNOS65+ variables of cognitive problems and depressed mood (Model 2), and finally all covariates were adjusted for (Model 3) to determine independent associations. All analyses were performed using Stata version 13.0(22). A threshold of $p<.05$ was considered significant.

For peer review only

Results

Sample size and characteristics

During the 11-year study period, a total of 6809 patients diagnosed with depression at age 65 or above were identified (Figure 1). Of these 899 patients were excluded as they already had a co-morbid diagnosis of dementia at the time (or within 3 months) of the late-life depression diagnosis. Of the remaining 5,910 patients diagnosed with late-life depression, 5104 (86.4%) did not receive a diagnosis of dementia in SLaM services during the study period (Supplementary Table 1), while 806 (13.6%) patients did.

The 806 patients with both a depression and dementia diagnosis in secondary care were included for final analysis. Baseline characteristics of this cohort are presented in Table 1. About a quarter has either a diagnosis of recurrent depression or had received a depression diagnosis before the index date. Following their depression diagnosis, the majority was prescribed antidepressant medication, but also 39% antipsychotics. Less than a quarter underwent CBT.

For peer review only

Table 1. Baseline characteristics 806 patients with late-life depression who later were diagnosed with dementia

Baseline characteristics	n (% or SD)
Mean age at depression diagnosis (years, SD)	78.3 (7.2)
Mean time in years from depression diagnosis to death/census (SD)	5.4 (3.0)
Female (%)	554 (68.7)
Non-white ethnicity (%)	156 (19.4)
Married or cohabiting (%)[a]	234 (29.6)
Mean index of deprivation (SD)[a]	26.7 (11.3)
Recurrent depressive disorder diagnosis (F33) or previous diagnosis of depression (%)	187 (23.2)
HoNOS65+ mental and physical health problems (%)[a,b]	
Agitated behaviour	128 (16.2)
Non-accidental self-injury	62 (7.9)
Drug/alcohol problems	35 (4.4)
Hallucinations or delusions	80 (10.2)
Depressed mood	488 (61.8)
Physical illness or disability	483 (61.1)
Cognitive problems	281 (35.7)
HoNOS65+ functional problems (%)[a,b]	
Activities of daily living	372 (47.2)
Living conditions	91 (11.7)
Occupational and recreational activities	272 (35.0)
Social relationships	173 (21.9)
Psychotropic use: in 2 years pre-depression diagnosis	
Antidepressant (%)	448 (55.6)
Psychotropic use: post-depression diagnosis	
Any antidepressant (%)	735 (91.2)
SSRI (%)	494 (61.3)
Antipsychotic (%)	310 (38.5)
Cognitive behavioural therapy	
Received CBT post-depression diagnosis (%)	179 (22.2)

[a]at the time of depression diagnosis
[b]Health of the Nation Outcome Scale 65+ (HoNOS65+) subscale scores 0-4 (0=least severe, 4 = most severe status) were categorised into scores of 0-1 representing that the patient was not experiencing problems in that domain, and scores of 2-4 representing experiencing problems. Values represent frequencies of patients scored as experiencing problems in that domain.

For peer review only

Predictors of accelerated dementia diagnosis

The average time from depression diagnosis to dementia diagnosis in this cohort was 2.8 years (SD=2.4). Of this group, 353 (43.8%) patients developed Alzheimer's disease (ICD-10 code F00), 199 (24.7%) vascular dementia (F01), 51 (6.3%) dementia in other diseases (F02) and 203 (25.2%) unspecified dementia (F03).

Table 2 presents the findings from a series of Cox proportional hazards multivariate models examining the associations of covariates with the time to dementia diagnosis. Analyses were first adjusted for the demographic factors of age, gender and deprivation (Model 1). Older age, non-white ethnicity and deprivation, as well as the HoNOS65+ subscale scores relating to problems with depressed mood, physical illness, cognition, ADL's and occupational/recreational activities, were significantly associated with faster progression to dementia. Female gender, a diagnosis of recurrent depression, prescription of antidepressants in the 2 years prior to depression diagnosis, CBT or prescription of antipsychotic drugs post-depression diagnosis, were all associated with slower progression to dementia.
Older age, deprivation, male gender and experiencing problems with ADLs remained significantly associated with faster progression to dementia when HoNOS65+ cognitive problems and depressed mood (Model 2) were entered into the model. Having a recurrent depressive disorder, being treated with antipsychotics or CBT remained protective.

In a model fully adjusted for all covariates (Model 3); older age, deprivation, and experiencing problems with cognition or depressed mood at the time of late-life depression diagnosis remained independently and significantly associated with a shorter time to dementia diagnosis. The associations of female gender, and a diagnosis of recurrent depression with slower progression to dementia diagnosis persisted. Adjustment for all covariates had an attenuating effect on the associations of CBT, antipsychotics and ADL problems with progression to dementia, which were no longer substantial predictors (see Figure 2).

Figure 3 represents mean time difference between depression and dementia diagnosis according to features present at depression diagnosis. On average patients with ADL problems developed dementia 10 months earlier and those with cognitive

For peer review only

problems 1.4 years earlier, while those with recurrent depression developed dementia 1.1 years later and those receiving CBT 11 months later. Table 3 shows time to dementia development according to severity of cognitive and mood problems rated by clinicians on the HoNOS65+ subscales. Those scored to have no or minor cognitive problems transitioned to dementia in 3-4 years, while those with moderate to severe in less than 2 years.

For peer review only

Table 2. Unadjusted and adjusted analyses of associations between covariates and time from late-life depression diagnosis to dementia diagnosis (Association with time to dementia diagnosis – hazard ratio (95% CI)).

Covariate	**Model 1** Adjusted for age, sex, deprivation	**Model 2** Model 1 + depressed mood[b] + cognitive problems[b]	**Model 3** Fully adjusted for all covariates
Age (per year increment)[a]	1.03 (1.02-1.04)***	1.03 (1.02-1.04)***	1.03 (1.02-1.04)***
Female sex	0.78 (0.67-0.91)**	0.76 (0.66-0.89)**	0.76 (0.64-0.89)**
Non-white ethnicity	1.23 (1.03-1.48)*	1.21 (1.01-1.46)*	1.14 (0.94-1.38)
Married or cohabiting status[a]	1.03 (0.87-1.21)	1.06 (0.90-1.25)	1.04 (0.88-1.24)
Deprivation (per 10 unit increase in IMD score)[a]	1.07 (1.00-1.14)*	1.08 (1.01-1.15)*	1.08 (1.02-1.16)*
Recurrent depressive disorder diagnosis (F33) or previous diagnosis of depression	0.72 (0.60-0.85)***	0.76 (0.64-0.91)**	0.79 (0.65-0.95)*
HoNOS65+ mental and physical health problems[a,b]			
Agitated behaviour	1.08 (0.89-1.32)	0.92 (0.76-1.13)	0.97 (0.78-1.21)
Non-accidental self-injury	0.85 (0.65-1.11)	0.73 (0.56-0.96)*	0.87 (0.65-1.16)
Drug/alcohol problems	1.35 (0.95-1.92)	1.30 (0.92-1.85)	1.20 (0.83 – 1.72)
Hallucinations and delusions	0.85 (0.67-1.08)	0.76 (0.60-0.97)*	0.83 (0.64-1.08)
Depressed mood	1.29 (1.11-1.49)**	1.23 (1.06-1.43)**	1.31 (1.11-1.54)**
Physical illness or disability	1.18 (1.02-1.37)**	1.12 (0.96-1.30)	0.95 (0.80-1.12)
Cognitive problems	1.79 (1.54-2.09)***	1.76 (1.51-2.05)***	1.65 (1.39-1.95)***
HoNOS65+ functional problems[a,b]			
Activities of daily living	1.44 (1.25-1.67)***	1.22 (1.05-1.43)*	1.18 (0.99-1.40)
Living conditions	1.04 (0.83-1.30)	0.90 (0.72-1.13)	0.83 (0.65-1.06)
Occupational/recreational activities	1.25 (1.08-1.46)**	1.14 (0.97-1.34)	1.18 (0.99-1.40)
Social relationships	1.12 (0.94-1.34)	1.04 (0.87-1.24)	1.03 (0.85-1.26)
Psychotropic use: in 2 years pre-depression diagnosis			
Antidepressant	0.85 (0.74-0.98)*	0.88 (0.76-1.02)	0.95 (0.81-1.11)
Psychotropic use: post-depression diagnosis			
Any antidepressant	0.95 (0.74-1.23)	0.93 (0.71-1.22)	1.09 (0.81-1.47)
SSRI	0.92 (0.80-1.07)	0.88 (0.76-1.02)	0.87 (0.74-1.03)
Antipsychotic	0.80 (0.69-0.92)**	0.77 (0.66-0.90)**	0.86 (0.73-1.03)
Cognitive behavioural therapy			
Received CBT post-depression diagnosis	0.74 (0.62-0.89)**	0.74 (0.62-0.89)**	0.89 (0.73-1.08)

***p<.001 ** p<.01 * p<.05
[a]at the time of depression diagnosis
[b]Health of the Nation Outcome Scale 65+ (HoNOS65+) subscale scores 0-4 (0=least severe, 4=most severe status) were categorised into scores of 0-1 representing that the patient was not experiencing problems in that domain, and scores of 2-4 representing experiencing problems. Hazard ratios were calculated based on scoring for experiencing problems in that domain.

For peer review only

Table 3. Mean time from depression diagnosis to dementia diagnosis per score increase on HoNOS65+ subscales at the time of depression diagnosis.

HoNOS65+ Scores	Mean time from depression diagnosis to dementia diagnosis in years (95% CI)
Cognitive Problems	
0 = no problem	3.77 (3.44-4.11)
1 = minor problem requiring no action	2.94 (2.67-3.20)
2 = mild problem but definitely present	2.05 (1.75-2.35)
3 = moderately severe problem	1.81 (1.39-2.22)
4 = severe to very severe problem	1.36 (0.25-2.46)
Depressed Mood	
0 = no problem	3.27 (2.80-3.74)
1 = minor problem requiring no action	3.08 (2.73-3.43)
2 = mild problem but definitely present	2.63 (2.34-2.93)
3 = moderately severe problem	2.66 (2.33-2.99)
4 = severe to very severe problem	2.46 (1.73-3.19)

For peer review only

Discussion

This study shows that in a cohort of older adults seen in secondary mental health services for depression and later diagnosed with dementia, shorter time to diagnosis of dementia was predicted by older age, male gender, new-onset depression, severity of depressive and cognitive symptoms, and living in a more deprived area.

Our findings regarding demographic features are in line with the current literature, with the majority of studies pointing towards an increased risk of dementia in depressed males(10, 23). Although social deprivation is commonly associated with poor engagement with, and underuse of mental health services(24), in our study patients from more deprived areas received a dementia diagnosis sooner; one plausible explanation for this being lower education levels and thus a worse cognitive reserve in this group(25).

Regarding the potential benefits of antidepressant medication, we found no substantial associations between the prescribing of antidepressant drugs in general, or specifically SSRIs, and time to dementia diagnosis. However, over 90% of the sample were prescribed antidepressant drugs following their depression diagnosis, and hence the analysis lacked a matched comparison group. Other studies of cohorts of subjects with late-life depression and mild cognitive impairment (MCI)(26), or without cognitive impairment(9) and more balanced levels of antidepressant use have, similarly, not detected associations with dementia risk. It has previously been suggested that poor response to antidepressant drugs in patients with MCI can be an indication of an especially increased risk of developing dementia(27), and patients in our study might have been referred to secondary care for this reason (as suggested by the fact that over half had used antidepressants in the past). Robust analyses of the efficacy of specific pharmacotherapies are often outside the scope of observational cohort studies, and controlled trials are yet to identify any candidate antidepressant treatments that can improve cognitive outcomes in older adults with depression(28, 29), except for some small short-term improvements in executive functioning(30, 31).

There is ongoing debate as to whether recurrent depression(32, 33), or history of

depression(34) carry an increased risk of dementia. Similar to the Whitehall II cohort study(8), we found that a diagnosis of recurrent depressive disorder was protective, but in relation to time to dementia diagnosis rather than overall dementia risk. We postulate that patients with a diagnosis of recurrent depression may have been more likely to be suffering initially from mood disorder distinct from incipient dementia, and hence their development of dementia came later. Patients in our study who were categorised as having new-onset depression may have followed a trajectory of steadily increasing depressive symptoms, which have previously been shown to characterise prodromal dementia(6-8), and reached a threshold of severity, at which point they reached secondary mental health services, and a diagnosis of depression was made. Methodological differences make it difficult to draw comparisons between findings relating to this relationship, as studies vary in their classifications of new-onset and recurrent depression, the time frame, and whether it is self-reported or clinically diagnosed. In our study, the severity and persistence of depressive symptoms across the cohort warranted a referral to secondary mental health services. Over half of the subjects had been prescribed antidepressant drugs during the two years prior to their depression diagnosis. Our results are consistent with findings from another large, community-based cohort study, in showing that a recent episode of depression in later life is a strong predictor of progression to dementia(26).

It is not surprising that greater cognitive problems predicted that a diagnosis of dementia would be made sooner, as past studies have shown that recently active depression in combination with MCI confers particularly high risk for Alzheimer's disease (AD); 40% progressed to AD within an average follow-up time of just over 2 years(26). Similarly, in a cohort of older adults from German primary care services with elevated depressive symptoms, co-existence of marked cognitive deficits was a distinguishing prognostic marker of subsequent dementia within the 6-year follow-up period(35). Against this background, the authors discouraged the attribution of cognitive impairment in late-life depression to “depressive pseudodementia” as it can be a strong indication of incipient dementia that warrants further investigation, a concept supported by our study. A case-control sample from the PAQUID cohort demonstrated a similar emergence of depressive symptoms and cognitive and functional decline in the years preceding the dementia phase of AD(36). In that cohort, progression of cognitive decline was accompanied by depressive symptoms, and as

For peer review only

a consequence of continued worsening, independence in activities of daily living then began to deteriorate. We illustrated problems with activities of daily living to be predictive of dementia being diagnosed sooner, even when adjusted for levels of depressive and cognitive symptoms. These results further contribute to the evidence for the progressive prodromal phase of dementia in relation to cognitive, functional, and depressive symptoms.

Strengths and limitations

Strengths of this study include the large, population-based sample and the potentially long follow-up time. The main limitations are related to generalisability of findings in this selected cohort. To be included into the study, patients needed a secondary care diagnosis of depression and to survive until they received a dementia diagnosis. These findings might not readily be generalisable to patients with milder depressive symptoms treated in primary care or those not readily seeking a dementia diagnosis(37). In comparison to those not seeking a dementia diagnosis (see Supplementary Table 1, Group 2), those included in the study (Group 1) had longer survival time (they needed to survive to until a dementia diagnosis was given), were more likely to be older, female, non-cohabiting, living alone, experiencing more problems with cognition and less problems with self-harm, physical illness and depressed mood at the time of depression diagnosis. These patients were also more likely to have been prescribed antidepressant drugs in the two years preceding their diagnosis of late-life depression and following their diagnosis they were more likely to have been prescribed antipsychotic and antidepressant drugs, including SSRIs.

Consequently, our findings relate to the time taken to receive a diagnosis of dementia in SLaM services, rather than the time taken to develop the diagnosable symptoms of dementia. This duration is undoubtedly influenced by potential barriers to timely diagnosis; service, provider, patient and care-giver factors contribute to delayed dementia diagnosis(38). A review of the literature on missed and delayed diagnosis of dementia found numerous studies to suggest that symptom severity and degree of impairment are important predictors of diagnostic sensitivity(38).

For peer review only

Conclusions

The aim of our study was to highlight clinical markers of accelerated progression to dementia in late-life depression. A knowledge of the indicators of the preclinical phase of dementia may be crucial to aid advancement in the identification, and hence the management, of the disease at the earliest possible stage. As patients at the earliest stage of dementia are most likely to benefit from intervention to delay progression, future efforts to improve the detection of dementia should focus on recognizing subtle, early manifestations of disease. By examining the temporal relationships between characteristics within late-life depression and progression to dementia, in the context of South London secondary mental health service users, we have highlighted features that may indicate sooner progression to dementia. In older patients presenting with new-onset depression, and more severe depressive and cognitive symptoms, clinicians should suspect a diagnosis of dementia. It may be beneficial to closely monitor these patients for signs of cognitive deterioration suggestive of dementia, to facilitate the earliest possible detection and diagnosis. These patients might also form an interesting group for which novel pharmacological interventions could be developed.

For peer review only

Author Contributions:

GeP, CM and DA conceived the study. GeP, CM and GaP performed the analysis and interpreted the data. GeP and CM wrote the manuscript. NK, MS, DA, RS provided expertise and feedback. All authors critically revised the manuscript and approved the final version of the study.

For peer review only

References:

1. Byers AL, Yaffe K. Depression and risk of developing dementia. Nat Rev Neurol. 2011;7(6):323-31.

2. Cherbuin N, Kim S, Anstey KJ. Dementia risk estimates associated with measures of depression: a systematic review and meta-analysis. BMJ Open. 2015;5(12):e008853.

3. Ownby RL, Crocco E, Acevedo A, John V, Loewenstein D. Depression and risk for Alzheimer disease: systematic review, meta-analysis, and metaregression analysis. Archives of general psychiatry. 2006;63(5):530-8.

4. Diniz BS, Butters MA, Albert SM, Dew MA, Reynolds CF. Late-life depression and risk of vascular dementia and Alzheimer's disease: systematic review and meta-analysis of community-based cohort studies. Br J Psychiatry. 2013;202(5):329-35.

5. Livingston G, Sommerlad A, Orgeta V, Costafreda SG, Huntley J, Ames D, et al. Dementia prevention, intervention, and care. Lancet. 2017;390(10113):2673-734.

6. Kaup AR, Byers AL, Falvey C, Simonsick EM, Satterfield S, Ayonayon HN, et al. Trajectories of Depressive Symptoms in Older Adults and Risk of Dementia. JAMA Psychiatry. 2016;73(5):525-31.

7. Mirza SS, Wolters FJ, Swanson SA, Koudstaal PJ, Hofman A, Tiemeier H, et al. 10-year trajectories of depressive symptoms and risk of dementia: a population-based study. Lancet Psychiatry. 2016;3(7):628-35.

8. Singh-Manoux A, Dugravot A, Fournier A, Abell J, Ebmeier K, Kivimäki M, et al. Trajectories of Depressive Symptoms Before Diagnosis of Dementia: A 28-Year Follow-up Study. JAMA Psychiatry. 2017;74(7):712-8.

9. Almeida OP, Hankey GJ, Yeap BB, Golledge J, Flicker L. Depression as a modifiable factor to decrease the risk of dementia. Transl Psychiatry. 2017;7(5):e1117.

10. Mirza SS, de Bruijn RF, Direk N, Hofman A, Koudstaal PJ, Ikram MA, et al. Depressive symptoms predict incident dementia during short- but not long-term follow-up period. Alzheimers Dement. 2014;10(5 Suppl):S323-S9.e1.

11. Ismail Z, Smith EE, Geda Y, Sultzer D, Brodaty H, Smith G, et al. Neuropsychiatric symptoms as early manifestations of emergent dementia: Provisional diagnostic criteria for mild behavioral impairment. Alzheimers Dement. 2016;12(2):195-202.

12. Jorm AF. History of depression as a risk factor for dementia: an updated review. Aust N Z J Psychiatry. 2001;35(6):776-81.

13. Donovan NJ, Locascio JJ, Marshall GA, Gatchel J, Hanseeuw BJ, Rentz DM, et al. Longitudinal Association of Amyloid Beta and Anxious-Depressive Symptoms in Cognitively Normal Older Adults. Am J Psychiatry. 2018;175(6):530-7.

14. Panza F, Frisardi V, Capurso C, D'Introno A, Colacicco AM, Imbimbo BP, et al. Late-life depression, mild cognitive impairment, and dementia: possible continuum? Am J Geriatr Psychiatry. 2010;18(2):98-116.

15. Fernandes AC, Cloete D, Broadbent MT, Hayes RD, Chang CK, Jackson RG, et al. Development and evaluation of a de-identification procedure for a case register sourced from mental health electronic records. BMC medical informatics and decision making. 2013;13:71.

16. Perera G, Broadbent M, Callard F, Chang CK, Downs J, Dutta R, et al. Cohort profile of the South London and Maudsley NHS Foundation Trust Biomedical Research Centre (SLaM BRC) Case Register: current status and recent enhancement of an Electronic Mental Health Record-derived data resource. BMJ Open. 2016;6(3):e008721.

17. Cunningham H, Tablan V, Roberts A, Bontcheva K. Getting more out of biomedical documents with GATE's full lifecycle open source text analytics. PLoS computational biology. 2013;9(2):e1002854.

18. World Health O. International statistical classification of diseases and related health problems: World Health Organization; 2004.

For peer review only

19. Stewart R, Soremekun M, Perera G, Broadbent M, Callard F, Denis M, et al. The South London and Maudsley NHS Foundation Trust Biomedical Research Centre (SLAM BRC) case register: development and descriptive data. BMC Psychiatry. 2009;9:51.

20. Uk GOV. English indices of deprivation 2010. Accessed; 2016.

21. Burns A, Beevor A, Lelliott P, Wing J, Blakey A, Orrell M, et al. Health of the Nation Outcome Scales for elderly people (HoNOS 65+). Br J Psychiatry. 1999;174:424-7.

22. StataCorp LP. Stata: release 13-statistical software. College Station, TX. 2013.

23. da Silva J, Gonçalves-Pereira M, Xavier M, Mukaetova-Ladinska EB. Affective disorders and risk of developing dementia: systematic review. Br J Psychiatry. 2013;202(3):177-86.

24. O'Brien A, Fahmy R, Singh SP. Disengagement from mental health services. A literature review. Soc Psychiatry Psychiatr Epidemiol. 2009;44(7):558-68.

25. Ott A, Breteler MM, van Harskamp F, Claus JJ, van der Cammen TJ, Grobbee DE, et al. Prevalence of Alzheimer's disease and vascular dementia: association with education. The Rotterdam study. BMJ. 1995;310(6985):970-3.

26. Gallagher D, Kiss A, Lanctot K, Herrmann N. Depression and Risk of Alzheimer Dementia: A Longitudinal Analysis to Determine Predictors of Increased Risk among Older Adults with Depression. Am J Geriatr Psychiatry. 2018;26(8):819-27.

27. Modrego PJ, Ferrández J. Depression in patients with mild cognitive impairment increases the risk of developing dementia of Alzheimer type: a prospective cohort study. Arch Neurol. 2004;61(8):1290-3.

28. Nebes RD, Pollock BG, Houck PR, Butters MA, Mulsant BH, Zmuda MD, et al. Persistence of cognitive impairment in geriatric patients following antidepressant treatment: a randomized, double-blind clinical trial with nortriptyline and paroxetine. J Psychiatr Res. 2003;37(2):99-108.

29. Bhalla RK, Butters MA, Mulsant BH, Begley AE, Zmuda MD, Schoderbek B, et al. Persistence of neuropsychologic deficits in the remitted state of late-life depression. Am J Geriatr Psychiatry. 2006;14(5):419-27.

30. Butters MA, Becker JT, Nebes RD, Zmuda MD, Mulsant BH, Pollock BG, et al. Changes in cognitive functioning following treatment of late-life depression. Am J Psychiatry. 2000;157(12):1949-54.

31. Devanand DP, Pelton GH, Marston K, Camacho Y, Roose SP, Stern Y, et al. Sertraline treatment of elderly patients with depression and cognitive impairment. Int J Geriatr Psychiatry. 2003;18(2):123-30.

32. Dotson VM, Beydoun MA, Zonderman AB. Recurrent depressive symptoms and the incidence of dementia and mild cognitive impairment. Neurology. 2010;75(1):27-34.

33. Barnes DE, Yaffe K, Byers AL, McCormick M, Schaefer C, Whitmer RA. Midlife vs late-life depressive symptoms and risk of dementia: differential effects for Alzheimer disease and vascular dementia. Archives of general psychiatry. 2012;69(5):493-8.

34. Wetherell JL, Gatz M, Johansson B, Pedersen NL. History of depression and other psychiatric illness as risk factors for Alzheimer disease in a twin sample. Alzheimer Dis Assoc Disord. 1999;13(1):47-52.

35. Heser K, Bleckwenn M, Wiese B, Mamone S, Riedel-Heller SG, Stein J, et al. Late-Life Depressive Symptoms and Lifetime History of Major Depression: Cognitive Deficits are Largely Due to Incipient Dementia rather than Depression. J Alzheimers Dis. 2016;54(1):185-99.

36. Amieva H, Le Goff M, Millet X, Orgogozo JM, Pérès K, Barberger-Gateau P, et al. Prodromal Alzheimer's disease: successive emergence of the clinical symptoms. Ann Neurol. 2008;64(5):492-8.

37. Winblad B, Amouyel P, Andrieu S, Ballard C, Brayne C, Brodaty H, et al. Defeating Alzheimer's disease and other dementias: a priority for European science and society. Lancet Neurol. 2016;15(5):455-532.

For peer review only

38. Bradford A, Kunik ME, Schulz P, Williams SP, Singh H. Missed and delayed diagnosis of dementia in primary care: prevalence and contributing factors. Alzheimer Dis Assoc Disord. 2009;23(4):306-14.

For peer review only

Figure 1. Flowchart showing cohort generation and outcomes

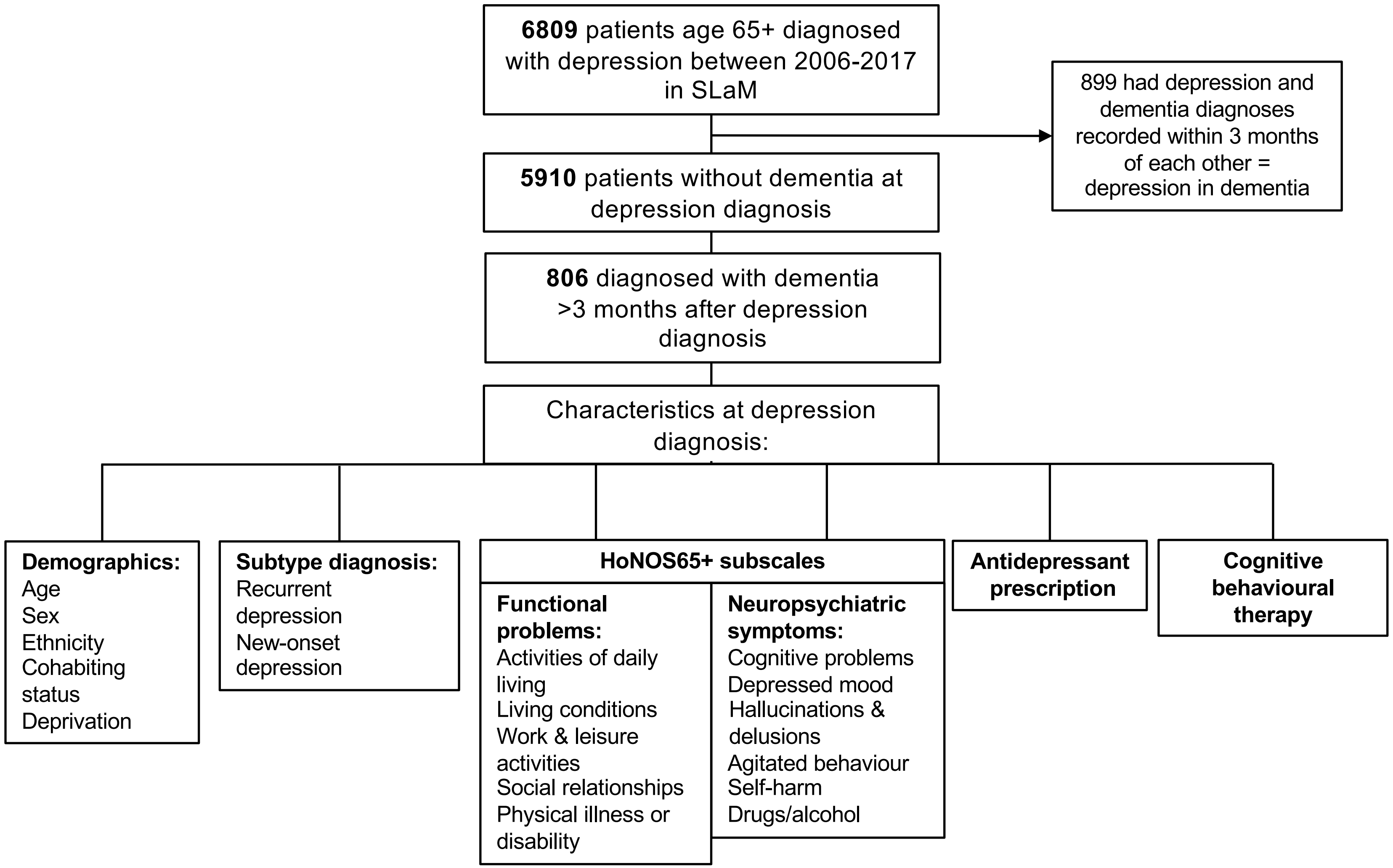

Figure 2. Plot of hazard ratios for accelerated conversion to dementia in a model adjusted for all co-variates

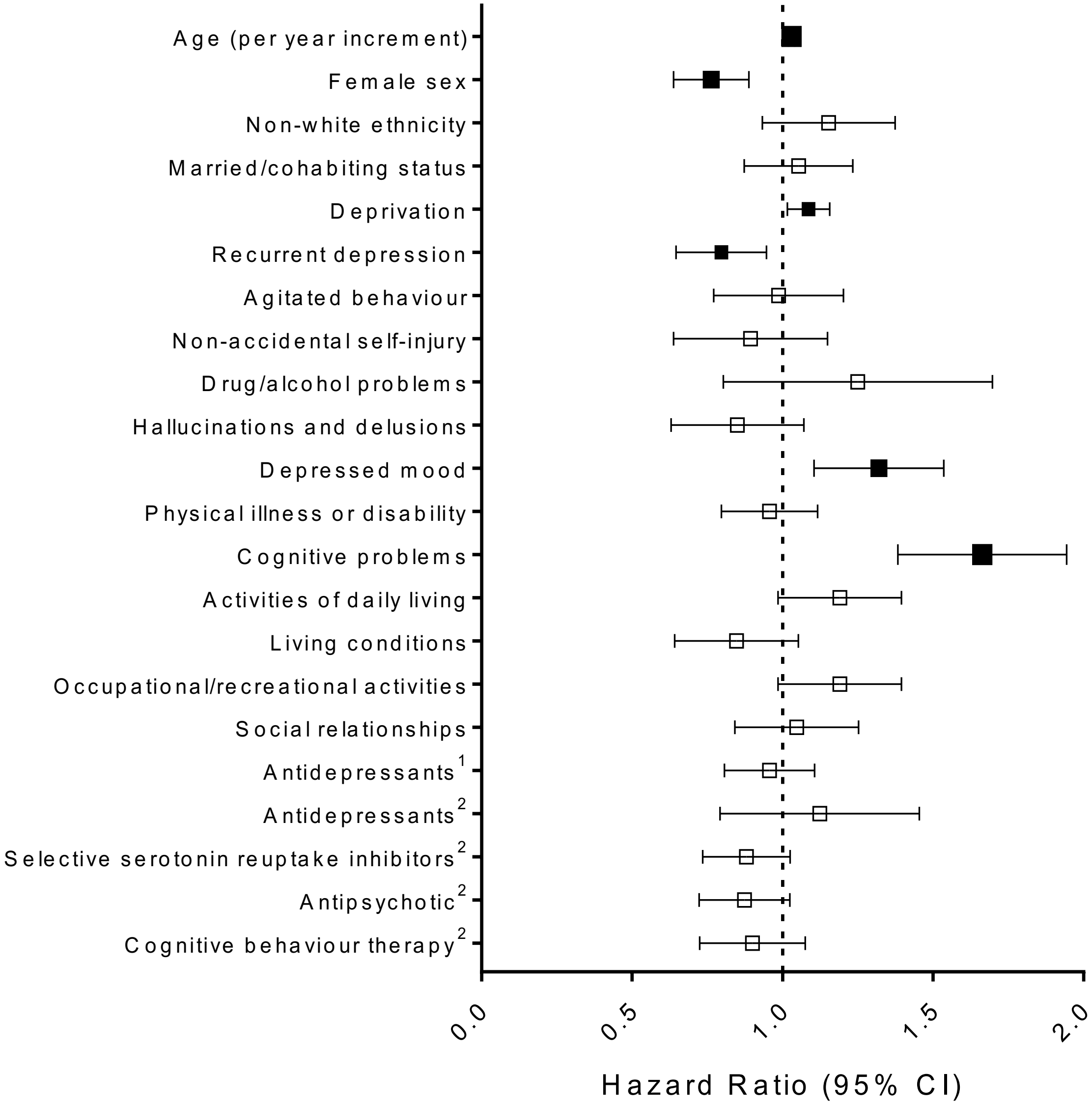

[1] in 2 years pre-depression diagnosis
[2] post-depression diagnosis

For peer review only - http://bmjopen.bmj.com/site/about/guidelines.xhtml

Figure 3. Impact of co-variates on time to dementia diagnosis in months

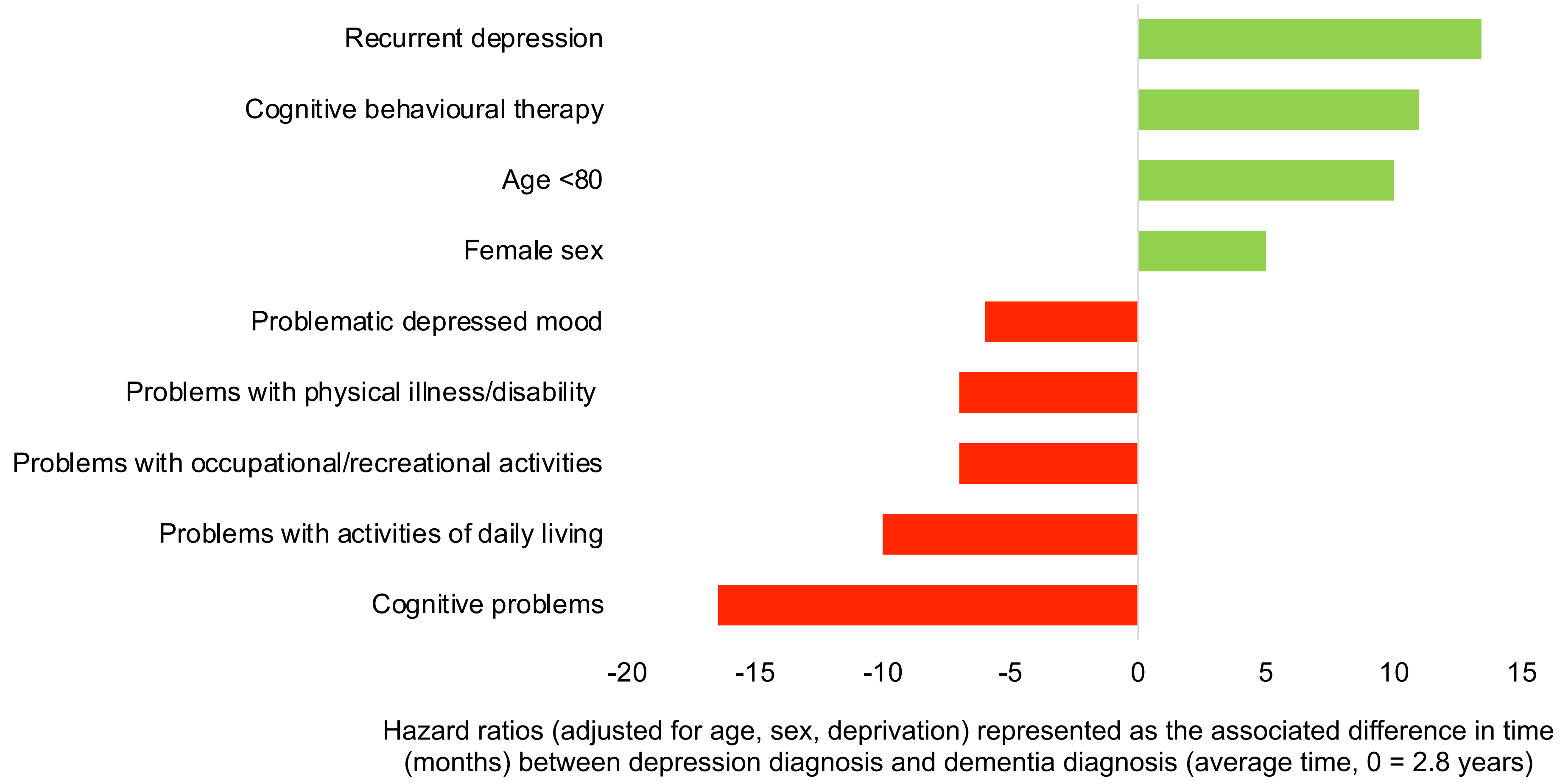

Supplementary Table 1. Baseline characteristics of the late-life depression sample (n=5910) according to dementia development.

Baseline characteristics	Dementia diagnosis >3 months after depression diagnosis	No dementia diagnosis recorded during follow-up	*p* value[a]
No. of patients	806	5104	
Mean age (SD)[b]	78.3 (7.2)	76.6 (8.1)	p<.001
Mean time in years from depression diagnosis to death/census (SD)	5.4 (3.0)	3.6 (3.3)	p<.001
Female (%)	554 (68.7)	3072 (60.2)	p<.001
Non-white ethnicity (%)	156 (19.4)	810 (16.6)	p=.050
Married or cohabiting status (%)[b]	234 (29.6)	1543 (33.3)	p=.041
Mean index of deprivation (SD)[b]	26.7 (11.3)	26.4 (11.9)	p=.545
F33 recurrent depressive disorder diagnosis (%)	187 (23.2)	1247 (24.4)	p=.449
HoNOS65+ mental and physical health problems (%)[b,c]			
Agitated behaviour	128 (16.2)	612 (13.9)	p=.092
Non-accidental self-injury	62 (7.9)	562 (12.8)	p<.001
Drug/alcohol problems	35 (4.4)	247 (5.6)	p=.174
Hallucinations or delusions	80 (10.2)	426 (9.7)	p=.712
Depressed mood	488 (61.8)	3054 (69.5)	p<.001
Physical illness or disability	483 (61.1)	3015 (68.6)	p<.001
Cognitive problems	281 (35.7)	818 (18.7)	p<.001
HoNOS65+ functional problems (%)[b,c]			
Activities of daily living	372 (47.2)	2211 (50.6)	p=.084
Living conditions	91 (11.7)	580 (13.5)	p=.171
Occupational and recreational activities	272 (35.0)	1582 (36.5)	p=.414
Social relationships	173 (21.9)	1029 (23.5)	p=.339
Psychotropic use: in 2 years pre-depression diagnosis			
Antidepressant (%)	448 (55.6)	2579 (50.5)	p=.008
Psychotropic use: post-depression diagnosis			
Antidepressant (%)	735 (91.2)	3683 (72.2)	p<.001
SSRI (%)	494 (61.3)	2175 (42.6)	p<.001
Antipsychotic (%)	310 (38.5)	1141 (22.4)	p<.001
Cognitive behavioural therapy			
Received CBT post-depression diagnosis (%)	179 (22.2)	839 (16.4)	p<.001

[a]χ^2 test, analysis of variance or independent samples t-test
[b]at the time of depression diagnosis
[c] Health of the Nation Outcome Scale 65+ (HoNOS65+) subscale scores 0-4 (0=least severe, 4 = most severe status) were categorised into scores of 0-1 representing that the patient was not experiencing problems in that domain, and scores of 2-4 representing experiencing problems. Values represent frequencies of patients scored as experiencing problems in that domain.

For peer review only

BMJ Open

Clinical factors associated with progression to dementia in people with late-life depression: a cohort study of patients in secondary care

Journal:	*BMJ Open*
Manuscript ID	bmjopen-2019-035147.R1
Article Type:	Original research
Date Submitted by the Author:	25-Feb-2020
Complete List of Authors:	Peakman, Georgia; IoPPN, Old Age Psychiatry;<br>Karunatilake, Nishshanka; South London and Maudsley NHS Foundation Trust<br>Seynaeve, Mathieu; South London and Maudsley NHS Foundation Trust; King's College London Institute of Psychiatry<br>Perera, Gayan; Institute of Psychiatry, Psychological Medicine<br>Aarsland, Dag; Stavanger University Hospital; King's College London Institute of Psychiatry, Department of Old Age Psychiatry<br>Stewart, Robert; King's College London, Institute of Psychiatry; South London and Maudsley NHS Foundation Trust<br>Mueller, Christoph; King's College London Institute of Psychiatry, Department of Old Age Psychiatry; South London and Maudsley NHS Foundation Trust
**Primary Subject Heading**:	Mental health
Secondary Subject Heading:	Geriatric medicine, Neurology
Keywords:	Depression & mood disorders < PSYCHIATRY, Dementia < NEUROLOGY, GERIATRIC MEDICINE, MENTAL HEALTH

SCHOLARONE™
Manuscripts

I, the Submitting Author has the right to grant and does grant on behalf of all authors of the Work (as defined in the below author licence), an exclusive licence and/or a non-exclusive licence for contributions from authors who are: i) UK Crown employees; ii) where BMJ has agreed a CC-BY licence shall apply, and/or iii) in accordance with the terms applicable for US Federal Government officers or employees acting as part of their official duties; on a worldwide, perpetual, irrevocable, royalty-free basis to BMJ Publishing Group Ltd ("BMJ") its licensees and where the relevant Journal is co-owned by BMJ to the co-owners of the Journal, to publish the Work in this journal and any other BMJ products and to exploit all rights, as set out in our licence.

The Submitting Author accepts and understands that any supply made under these terms is made by BMJ to the Submitting Author unless you are acting as an employee on behalf of your employer or a postgraduate student of an affiliated institution which is paying any applicable article publishing charge ("APC") for Open Access articles. Where the Submitting Author wishes to make the Work available on an Open Access basis (and intends to pay the relevant APC), the terms of reuse of such Open Access shall be governed by a Creative Commons licence – details of these licences and which Creative Commons licence will apply to this Work are set out in our licence referred to above.

Other than as permitted in any relevant BMJ Author's Self Archiving Policies, I confirm this Work has not been accepted for publication elsewhere, is not being considered for publication elsewhere and does not duplicate material already published. I confirm all authors consent to publication of this Work and authorise the granting of this licence.

For peer review only

Clinical factors associated with progression to dementia in people with late-life depression: a cohort study of patients in secondary care

Georgia Peakman[1], Nishshanka Karunatilake[2], Mathieu Seynaeve[1,2], Gayan Perera[1], Dag Aarsland[1,3], Robert Stewart[1,2], Christoph Mueller[1,2]

[1]King's College London, Institute of Psychiatry, Psychology and Neuroscience, London, UK
[2]South London and Maudsley NHS Foundation Trust, London, UK
[3]Centre for Age-Related Medicine, Stavanger University Hospital, Stavanger, Norway

Corresponding author: Georgia Peakman; King's College London, Institute of Psychiatry, Psychology and Neuroscience (IoPPN), De Crespigny Park, London, SE5 8AF, United Kingdom; email: georgia.peakman.18@ucl.ac.uk; phone: +44 207 848 0626.

Declarations of interest:

RS has received research funding from Roche, Pfizer, Janssen, Lundbeck and In-Silico-Bioscience. DA has received research support and/or honoraria from Astra-Zeneca, H. Lundbeck, Novartis Pharmaceuticals and GE Health, and serves as paid consultant for H. Lundbeck and Axovant. All other authors declare no conflict of interest.

Funding:

GeP received undergraduate bursaries from the Alzheimer's Society and the Wellcome Trust to conduct this project. CM, GaP, DA and RS receive salary support from the National Institute for Health Research (NIHR) Biomedical Research Centre at South London and Maudsley NHS Foundation Trust and King's College London, and RS is a NIHR Senior Investigator. The views expressed are those of the author(s) and not necessarily those of the NHS, the NIHR or the Department of Health.

For peer review only

Word count: 3,842

Tables: 3

Figures: 3

For peer review only

Clinical factors associated with progression to dementia in people with late-life depression: a cohort study of patients in secondary care.

Objectives: Depression can be a prodromal feature or a risk factor for dementia. We aimed to investigate which clinical factors in patients with late-life depression are associated with a higher risk of developing dementia and a more rapid conversion.

Design: Retrospective cohort study.

Setting: South London and Maudsley NHS Foundation Trust (SLaM) secondary mental healthcare services.

Participants: The SLaM Clinical Record Interactive Search (CRIS) was used to retrieve anonymised data on 3,659 patients aged 65 years or older who had received a diagnosis of depression in mental health services and had been followed-up for at least 3 months.

Outcome measures: Predictors of development of incident dementia were investigated, including demographic factors, health status rated on the Health of the National Outcome scale for older people (HoNOS65+), depression recurrence, and treatments including psychotropic drugs and cognitive behavioural therapy (CBT).

Results: In total, 806 (22.0%) patients developed dementia over a mean follow-up time of 2.7 years. Significant predictors of receiving a dementia diagnosis in fully adjusted models and after accounting for multiple comparisons were: older age (adjusted hazard ratio (HR)=1.06, 95% CI: 1.05-1.07 per year increase) and the HoNOS65+ subscales measuring cognitive problems (HR=4.46, 95% CI: 3.47-5.73 for scores in the problematic range). Recurrent depressive disorder or past depression (HR=0.55, 95% CI: 0.42-0.73) and the receipt of CBT (HR=0.73 95% CI: 0.61-0.87) were associated with a lower dementia risk.

Conclusions: In older adults with depression, a higher risk of being subsequently diagnosed with dementia was predicted by higher age, new onset depression, severity of cognitive symptoms and not receiving CBT. Further exploration is needed to determine whether the latter risk factors are responsive to interventions.

Key words: depression, dementia, diagnosis, predictors, secondary care.

For peer review only

Article Summary

Strengths and limitations of this study

- This study used a large naturalistic sample of older patients receiving routine mental healthcare, with access to anonymised clinical data.
- The sample was limited to patients who had been diagnosed with depression, followed-up, and if applicable, diagnosed with dementia within the same secondary provider; it is not readily generalisable to cases of depression treated in other settings.
- Time between depression diagnosis and dementia diagnosis is additionally influenced by potential barriers to seeking a dementia diagnosis such as service, provider, patient and care-giver factors, which were not captured by this analysis.
- The influences of other comorbid long-term conditions and resultant polypharmacy were not fully considered, and causal inferences cannot be readily made from observational data.

For peer review only

Introduction

A link between depression and dementia is well established, and growing evidence indicates that the timing of depression is important in defining the nature of the relationship [1]. Late-life depression is projected to increase substantially by 2050 [2] and has consistently been associated with up to a two-fold increased risk for dementia [3-5], and it is now recognised as a key potentially modifiable risk factor for the condition [6]. However, causality is yet to be established; it remains unclear whether, and in which cases, late-life depression is an independent risk factor for dementia or a prodromal symptom.

Identification of the clinical characteristics of older adults with depression that are indicative of incipient dementia would facilitate accurate classification of individuals at high risk for early progression and subsequently, timelier treatment**.** The pattern and severity of depressive symptoms in late life has emerged as one important factor in determining the likelihood of progression to dementia. In two large, longitudinal cohort studies of older adults, those subjects characterised by trajectories of high and steadily increasing depressive symptoms over several years were significantly more likely to develop dementia [7 8]. Having an episode of high depressive symptoms that remitted was not associated with greater dementia risk [8]. Similarly, recent data from the Whitehall II cohort study characterised those with late-life depression who later developed dementia as having a distinguishable trajectory of increasing depressive symptoms, emerging 11 years before dementia diagnosis [9]. Depressive symptoms during mid-life, even when recurrent, did not increase dementia risk in this cohort. These findings, in addition to other reports of graded associations with depression severity and raised dementia risk being contingent on the two being in closer temporal proximity [10 11], lend support to the hypotheses that depressive symptoms during later life can be an early manifestation of dementia [12], or that the two conditions share common aetiological factors [13 14]; potentially both are consequences of an underlying neurodegenerative process [15].

While it is well established that greater severity of depressive symptoms is closely associated with accelerated development of dementia, other associations relevant to

For peer review only

this relationship are less clear. Using naturalistic data from a cohort of secondary mental health service users, the present study examined associations between patient characteristics and interventions within late-life depression and the risk of progression to a diagnosis of dementia.

For peer review only

Methods

Source of data

Data for this study were obtained via the South London and Maudsley NHS Foundation Trust (SLaM), one of Europe's largest mental healthcare providers, serving 1.3 million residents in four south London boroughs (Croydon, Lambeth, Lewisham and Southwark), as well as delivering specialist national services. SLaM adopted fully electronic records across all of its services from 2006, having imported legacy systems which include a records system run for several years previously in dementia care. The Clinical Record Interactive Search (CRIS) application was developed in 2008 to provide access to de-identified structured and open-text data extracted from this electronic health records system, containing records on over 400,000 SLaM mental health service users [16 17]. CRIS has been enhanced by a range of natural language processing ('text-mining') applications [18] which extract structured data from text fields, including applications to ascertain pharmacotherapy [17]. CRIS has research ethical approval as an anonymised database for secondary analysis (Oxford REC C 08/H0606/71+5).

Sample

Using CRIS, people aged 65 or older who received a diagnosis of depression from SLaM services within the 11-year period between 01/01/2006 to 30/03/2017 were identified according to the International Statistical Classification of Diseases and Related Health Problems 10th Revision (ICD-10) [19] criteria described below. Date of first depression diagnosis after the age of 65 years served as the index date. Patients were followed-up until the date of their first dementia diagnosis (incident dementia) given in the health record; or their last face-to-face contact with a professional from secondary mental health services (clinical event or appointment); or death; or a censoring point on 30/06/2017. Patients were excluded if they had a diagnosis of dementia recorded before or within 3 months of the index date, or if they had less than 3 months of contact with SLaM services after their depression diagnosis.

For peer review only

Measurements

Cases of depression and dementia according to ICD-10 codes were ascertained, both from diagnoses recorded in structured fields and in free text within clinical correspondence, through a natural language processing algorithm [18].

Late-life depression

Initial depression diagnoses (the index contact) were determined by the following diagnostic codes from ICD-10 [19] within a primary or secondary diagnosis: F32 (depressive episode), F32.2 (severe depressive episode without psychotic symptoms), F32.3 (severe depressive episode with psychotic symptoms), F33 (recurrent depressive disorder), F33.2 (recurrent depressive disorder, current episode severe without psychotic symptoms), F33.3 (recurrent depressive disorder, current episode severe with psychotic symptoms). Data were also obtained for any depression diagnoses (F32, F33) recorded before the index date.

Dementia

Dementia diagnoses were determined by the following diagnostic codes from ICD-10[19] within a primary or secondary diagnosis: F00 (dementia in Alzheimer's disease), F01 (vascular dementia), F02 (dementia in other diseases classified elsewhere), F03 (unspecified dementia).

Covariates

Data were also extracted on age (at depression diagnosis), gender, ethnicity, marital or cohabiting status, local authority, a neighbourhood-level deprivation score (Index of Multiple Deprivation, 2010) [20], psychotropic medication (classified into antipsychotic, antidepressant and selective serotonin reuptake inhibitor (SSRI) types) prescribed in the period 2 years prior to depression diagnosis and following diagnosis, and the receipt of cognitive behaviour therapy (CBT) after depression diagnosis. The Health of the Nation Outcome Scales for older people [21] (HoNOS65+) is routinely administered in SLaM services, and the subscale scores available closest to the depression diagnosis extracted for this analysis were: agitated behaviour, non-accidental self-injury, drug/alcohol problems, hallucinations or delusions, depressed mood, physical illness or disability, cognitive problems, activities of daily living (ADLs), living conditions, occupational and recreational activities, social relationships.

For peer review only

HoNOS65+ items are rated from 0 (least severe status) to 4 (most severe status). For the purpose of this analysis scores of 0-1 were considered to indicate that the patient was not experiencing problems in the domain, and scores of 2-4 were considered to indicate experiencing severe problems as rated by the clinician.

Statistical Analysis

Baseline characteristics of the cohort grouped according to dementia development (those who were diagnosed with dementia within secondary mental health services and those who didn't have a dementia diagnosis at their last contact with services), were compared using chi-square tests for categorical variables, and independent samples t-tests or analysis of variance (ANOVA) for continuous variables.

Cox proportional hazards regression models were used to analyse associations between the predictor variables at depression diagnosis and hazard of receiving a diagnosis of dementia. Multivariate models were first adjusted for demographic factors; age, gender and deprivation (Model 1), then for the HoNOS65+ variables of cognitive problems and depressed mood (Model 2), and finally all variables were adjusted for in Model 3 to determine independent associations. All analyses were performed using Stata version 13.0 [22]. We assessed the proportional-hazard assumptions for each variable on the basis of Schoenfeld residuals and included variables as time-varying if they were violated. We report both a significance level of $p<.05$ and a Bonferroni adjusted $p<.002$ to account for multiple comparisons. In a second step, we only considered those patients in the cohort who had a diagnosis of dementia recorded, to determine how the duration from depression to dementia diagnosis varied according to the predictors that had yielded significance in the above Cox regression models. As 19% of the cohort had missing data on at least one covariate, we created 19 imputed datasets by replacing missing values through simulated values assembled from potential covariates and outcome values [23 24].

Patient and public involvement

A patient-led committee provides operational oversight of the CRIS resource and ensures that the proposed study has both scientific value as well as benefit for patients and carers [17 25]. The authors had applied to the oversight committee with an outline of the proposed analysis, including data linkages, and the project was

approved on 31/05/2017. There was no further patient and public involvement in this register-based study.

For peer review only

Results

Sample size and characteristics

During the 11-year study period, a total of 6,670 patients diagnosed with depression at age 65 or above were identified (Figure 1). Of these, 887 patients were excluded because they already had a co-morbid diagnosis of dementia at the time or within 3 months of the depression diagnosis. A further 2,124 patients were excluded because they were followed-up for less than 3 months after their depression diagnosis. The final study cohort consisted of 3,659 patients with late-life depression, of which 806 (22.0%) received a diagnosis of dementia from mental health services during the follow-up period. Mean follow-up time until dementia diagnosis, last clinical face-to-face contact, census or death was 2.7 years (SD +/- 2.6 years); and this did not differ significantly between those who were or were not diagnosed with dementia (see Table 1).

Baseline characteristics of the late-life depression cohort as a whole and according to dementia development are presented in Table 1. Patients who were subsequently diagnosed with dementia were significantly older and more likely to be female, have cognitive problems and impairment of their activities of daily living (ADLs). They were less likely to have recurrent depression, non-accidental self-injury recorded, or problems related to their depressed mood or social relationships. In terms of pharmacotherapy, those who were diagnosed with dementia were more likely to be prescribed any antidepressant, a selective serotonin reuptake inhibitor (SSRI), or an antipsychotic after depression diagnosis, but were less likely to have received cognitive behavioural therapy (CBT).

For peer review only

Table 1: Baseline characteristics of the late-life depression cohort according to whether a dementia diagnosis was established.

Baseline characteristics	Whole cohort	Dementia diagnosis >3 months post depression diagnosis	No dementia diagnosis during follow-up	*p*-value[a]
No. of patients	3,659	806	2,853	
Mean age (SD)[b]	76.0 (7.7)	78.3 (7.2)	75.3 (7.7)	<.001
Mean time in years from depression diagnosis to census (dementia/death/last clinical contact) (SD)	2.7 (2.6)	2.8 (2.4)	2.7 (2.6)	.365
Female (%)	64.1	68.7	62.7	.002
Non-white ethnicity (%)	17.6	19.4	17.1	.121
Married or cohabiting status (%)[b]	32.1	29.6	32.8	.091
Mean index of deprivation (SD)[b]	27.0 (11.5)	26.7 (11.3)	27.0 (11.5)	.487
F33 recurrent depressive disorder diagnosis (%)	28.5	23.2	30.0	<.001
**HoNOS65+ mental and physical health problems (%)[b,c]**				
Agitated behaviour	15.9	16.2	15.8	.775
Non-accidental self-injury	12.4	7.9	13.8	<.001
Drug/alcohol problems	5.6	4.4	6.0	.100
Hallucinations or delusions	11.3	10.2	11.7	.232
Depressed mood	67.4	61.8	69.1	<.001
Physical illness or disability	60.8	61.1	60.8	.846
Cognitive problems	19.1	35.7	14.1	<.001
**HoNOS65+ functional problems (%)[b,c]**				
Activities of daily living	44.1	47.2	43.1	.042
Living conditions	13.1	11.7	13.6	.168
Occupational and recreational activities	36.0	35.0	36.3	.526
Social relationships	25.2	21.9	26.2	.015
**Psychotropic use: in 2 years pre depression diagnosis**				
Antidepressant (%)	56.1	55.6	56.3	.720
**Psychotropic use: post depression diagnosis**				
Antidepressant (%)	86.5	91.2	85.1	<.001
SSRI (%)	55.2	61.3	53.4	<.001
Antipsychotic (%)	34.4	38.5	33.2	.006
**Cognitive behavioural therapy**				
Received CBT post-depression diagnosis (%)	26.8	22.2	28.1	.001

[a] χ^2 test, analysis of variance or independent samples t-test
[b] at or closest to the time of depression diagnosis
[c] Health of the Nation Outcome Scale 65+ (HoNOS65+) subscale scores 0-4 (0=least severe, 4 = most severe status). Values represent frequencies of patients scored as experiencing problems (score 2-4) in that domain.

For peer review only

Predictors of receiving a dementia diagnosis in secondary care services

Table 2 presents the findings from a series of multivariate Cox proportional hazards models examining associations between the predictors in late-life depression and risk of dementia diagnosis. Analyses were first adjusted for the demographic factors of age, gender and deprivation (Model 1). In this model, when considering a *p*-value of $p<.002$ as significant to account for multiple comparisons, older age and non-white ethnicity, as well as the HoNOS65+ subscale scores linked to problems with physical illness, cognition and ADLs were significantly associated with a higher risk of developing dementia, while recurrent depression and treatment with CBT were associated with lower dementia risk. In Model 2 and the fully adjusted Model 3, at a significance level of $p<.002$, older age and cognitive problems remained associated with progression to dementia, and recurrent depressive disorder and treatment with CBT both remained associated with lower dementia risk.

Figure 2 presents the hazard ratios generated from Model 3 which was fully adjusted for all predictor variables, applying a significance level of $p<.05$. A lower risk of dementia diagnosis was detected in those presenting with non-accidental self-injury and hallucinations and/or delusions, while those prescribed any antidepressant had a 34% increased dementia risk. Adding age as a squared term to the model didn't affect the predictors yielding significance, with the exception of non-accidental self-injury (attenuated to a trend; $p=.076$).

For peer review only

Table 2: Results from a series of multivariate Cox proportional hazards models analysing associations between predictor variables and risk of dementia diagnosis (adjusted hazard ratio (95% CI)) in 3,659 patients with late-life depression, of whom 806 were subsequently diagnosed with dementia during study follow-up.

**Predictor variables**	**Model 1** Adjusted for age, gender, deprivation	**Model 2** Model 1 + depressed mood[a,b] + cognitive problems[a,b]	**Model 3** Adjusted for all predictors
Age (per year increment)[a]	1.07 (1.06-1.08)**	1.06 (1.05-1.07)**	1.06 (1.05-1.07)**
Female gender	0.95 (0.82-1.11)	0.97 (0.83-1.12)	0.99 (0.84-1.16)
Non-white ethnicity	1.36 (1.13-1.62)**	1.22 (1.02-1.46)*	1.15 (0.96-1.39)
Married or cohabiting status[a]	1.07 (0.91-1.25)	1.10 (0.94-1.29)	1.09 (0.92-1.28)
Deprivation (per 10 unit increase in IMD score)[a]	1.01 (0.95-1.08)	0.99 (0.94-1.06)	0.99 (0.93-1.05)
Recurrent depressive disorder diagnosis (F33) or previous diagnosis of depression	0.47 (0.36-0.62)**	0.52 (0.39-0.69)**	0.55 (0.42-0.73)**
**HoNOS65+ mental and physical health problems[a,b]**			
Agitated behaviour	1.09 (0.90-1.33)	0.87 (0.71-1.06)	0.96 (0.78-1.18)
Non-accidental self-injury	0.69 (0.53-0.88)*	0.68 (0.53-0.89)*	0.77 (0.59-0.99)*
Drug/alcohol problems	1.03 (0.72-1.48)	0.97 (0.67-1.40)	1.08 (0.75-1.54)
Hallucinations and delusions	0.88 (0.70-1.10)	0.71 (0.56-0.90)*	0.74 (0.58-0.95)*
Depressed mood	1.11 (0.96-1.28)	1.01 (0.88-1.18)	1.06 (0.90-1.24)
Physical illness or disability	1.33 (1.15-1.54)**	1.18 (1.02-1.38)*	1.10 (0.93-1.29)
Cognitive problems	4.89 (3.85-6.21)**	4.88 (3.84-6.21)**	4.46 (3.47-5.73)**
**HoNOS65+ functional problems[a,b]**			
Activities of daily living	1.64 (1.32-2.05)**	1.14 (0.91-1.44)	1.05 (0.82-1.33)
Living conditions	1.04 (0.83-1.31)	0.90 (0.71-1.14)	0.89 (0.69-1.13)
Occupational/recreational activities	1.13 (0.97-1.32)	1.02 (0.87-1.19)	1.06 (0.89-1.26)
Social relationships	1.10 (0.85-1.42)	1.04 (0.80-1.35)	1.10 (0.84-1.44)
**Psychotropic use: in 2 years predepression diagnosis**			
Antidepressant	0.87 (0.76-1.00)*	0.90 (0.78-1.03)	0.93 (0.80-1.07)
**Psychotropic use: post depression diagnosis**			
Any antidepressant	1.21 (0.95-1.55)	1.27 (1.00-1.62)	1.34 (1.02-1.75)*
SSRI	1.12 (0.97-1.29)	1.11 (0.97-1.29)	1.06 (0.91-1.24)
Antipsychotic	0.88 (0.76-1.02)	0.85 (0.73-0.98)*	0.98 (0.84-1.14)
**Cognitive behavioural therapy**			
Received CBT post-depression diagnosis	0.62 (0.52-0.73)**	0.69 (0.58-0.82)**	0.73 (0.61-0.87)**

*$p<.05$
**$p<.002$ (Bonferroni corrected p-value)
[a] at or closest to the time of depression diagnosis
[b] Health of the Nation Outcome Scale 65+ (HoNOS65+) subscale scores 0-4 (0=least severe, 4 = most severe status). Values represent frequencies of patients scored as experiencing problems (score 2-4) in that domain.

For peer review only

Characteristics of 806 patients who received a dementia diagnosis

In those who received a dementia diagnosis, the average time from depression diagnosis to dementia diagnosis was 2.8 years (SD +/- 2.4). Of this group, the first recorded dementia subtype was Alzheimer's disease for 353 (43.8%), vascular dementia for 199 (24.7%), dementia in in other diseases for 51 (6.3%), and unspecified dementia for 203 (25.2%) patients.

Figure 3 represents variations in the mean time between depression and dementia diagnosis according to the factors which were significantly associated with dementia risk in the fully adjusted Model 3, after accounting for multiple comparisons. An age above 78 years at the point of depression diagnosis translated to receiving a dementia diagnosis 9 months earlier than average. Presenting with cognitive problems recorded on the HoNOS65+ instrument translated to a dementia diagnosis almost 17 months sooner than average. Table 3 shows time to dementia diagnosis according to severity of cognitive problems rated by clinicians on the HoNOS65+ subscale. Those who were scored to have no or minor cognitive problems at the time of their depression diagnosis progressed to dementia in 3-4 years, and those with moderate to severe problems in under 2 years. Having a diagnosis of recurrent depressive disorder or a previous diagnosis of depression were associated with a dementia diagnosis almost 14 months later than average, and treatment with CBT to 11 months later.

For peer review only

Table 3: Mean time from depression diagnosis to dementia diagnosis per score increase on the HoNOS65+ cognitive problems subscale recorded at the time of depression diagnosis, in 806 patients who were subsequently diagnosed with dementia.

HoNOS65+ cognitive problems scores	Mean time from depression diagnosis to dementia diagnosis in years (95% CI)
0 = no problem	3.77 (3.44 - 4.11)
1 = minor problem requiring no action	2.94 (2.67 - 3.20)
2 = mild problem but definitely present	2.05 (1.75 - 2.35)
3 = moderately severe problem	1.81 (1.39 - 2.22)
4 = severe to very severe problem	1.36 (0.25 - 2.46)

For peer review only

Discussion

This study showed in a cohort of over 3,500 older adults seen in secondary mental health services for depression that presenting at an older age, with new-onset depression and more severe cognitive problems, and not being treated with CBT, were each predictive of being subsequently diagnosed with dementia.

There is ongoing debate as to whether recurrent depression [26 27], or history of depression [28] carry an increased risk of dementia. We found that a diagnosis of recurrent depressive disorder was protective in relation to overall dementia risk, and in those who went on to develop dementia, recurrent depression was associated with a slower progression to a diagnosis which translated to over a year later than the average time. We postulate that the 187 patients with recurrent depression who developed dementia (23.2% of the total 806 patients who developed dementia) were more likely to be suffering initially from mood disorder distinct from incipient dementia, and so development of dementia overall came later. The remaining patients who were categorised as having new-onset depression may have been following a trajectory of newly emergent, steadily increasing depressive symptoms which have previously been shown to characterise prodromal dementia [7-9]; and reached a threshold of severity, at which point they reached secondary mental health services and the index diagnosis of depression was made. Methodological differences complicate comparisons between findings relating to the relationship between recurrent depression and dementia risk, as studies vary in their classifications of new-onset and recurrent depression, the time frame, and whether it is self-reported or clinically diagnosed. In this study, the severity and persistence of clinical symptoms across the cohort warranted a referral to secondary mental health services. The results are consistent with the findings from another large, community-based cohort study, in showing that a recent episode of depression in later life is a strong predictor of progression to dementia [29].

It is not surprising that more severe cognitive impairment in late-life depression was associated with a higher dementia risk and predicted that a diagnosis of dementia would be made sooner. Past studies have shown that recently active depression in combination with MCI confers particularly high risk for Alzheimer's disease; 40%

progressed to Alzheimer's disease within an average follow-up time of just over 2 years [29]. Similarly, in a cohort of older adults with high depressive symptoms in German primary care services, co-existence of marked cognitive deficits was a distinguishing prognostic marker of subsequent dementia within the 6-year follow-up period [30]. Against this background, the authors discouraged the attribution of cognitive impairment in late-life depression to "depressive pseudodementia" as it can be a strong indication of incipient dementia that warrants further investigation; a concept supported by our study. A case-control sample from the PAQUID cohort demonstrated a similar emergence of depressive symptoms and cognitive and functional decline in the years preceding the dementia phase of AD [31]. In that cohort, progression of cognitive decline was accompanied by depressive symptoms, and as a consequence of continued worsening, independence in activities of daily living then began to deteriorate.

Regarding the potential effects of antidepressant medication, although not significant after accounting for multiple comparisons, those prescribed 'any antidepressant' appeared to be at a higher risk of developing dementia in our cohort. This is in line with the growing evidence for this association from observational studies [32], and is consistent with a large, matched, prospective cohort study that found that antidepressant monotherapy was associated with an over two-fold increased dementia risk in the over 60s population after adjusting for multiple confounders (including depression) [33]. It's likely that a number of patients in the study were referred to secondary care following treatment failure, as suggested by the fact that over half had been prescribed antidepressants prior to reaching the service. Poor antidepressant response has been reported to be indicative of an especially increased risk of dementia in patients with cognitive impairment [34], however meta-analyses have suggested that the efficacy of antidepressants might generally be reduced in patients aged 65 and older [35 36]. As over 85% of our cohort were prescribed antidepressant drugs following their depression diagnosis, the analysis lacked the matched comparison group required to ascertain whether this indeed influenced dementia risk. Also, as only a crude measure of depression severity was adjusted for, it's possible that the few patients not prescribed an antidepressant might have suffered from milder forms of depression, meaning that the association with dementia risk reflects differences in depression symptomology rather than antidepressant treatment.

For peer review only

Potentially of note is that the ‘any antidepressant’ group yielded an increased dementia risk, but the SSRI group did not. This could be due to a lack of statistical power as the number of patients prescribed specifically SSRIs was lower, or it could indicate that the inclusion of antidepressants with a higher anticholinergic burden in this group (e.g. tricyclic antidepressants) might have contributed to the increased hazard of developing dementia [37]. Robust analyses of the efficacy of specific pharmacotherapies are often outside the scope of observational cohort studies, and from our study we cannot infer any neuroprotective effects of antidepressants. Controlled trials are yet to identify any candidate antidepressant treatments that can improve cognitive outcomes in older adults with depression [38 39], except for some small short-term improvements in executive functioning [40 41].

After adjusting for multiple confounders, undergoing CBT was associated with an almost 30% reduced risk of later being diagnosed with dementia. One likely explanation is that the patients who were offered CBT systematically differed from those not offered the intervention beyond what was possible to control for through adjusting for confounders, particularly in relation to their severity of depressive and cognitive symptoms. On the other hand, cognitive behavioural techniques are known to reduce some of the typical risk factors for dementia such as lack of cognitive, physical or social activity, a restricted diet or sleep disorders [42 43]. Further, CBT for insomnia has shown benefits to executive functioning in a small randomized study of people with MCI and insomnia [44]. Whether CBT needs to be adapted for late-life depression and could show benefits to cognitive decline requires further exploration [45].

Of note, although not considered significant after adjustment for multiple comparisons, the presence of non-accidental self-injury and psychotic symptoms appeared to be associated with a lower dementia risk. As multiple comparisons are considered conservative methods in Cox analysis [46], these results need to be given sufficient consideration. They are consistent with a previous study of late-life depression that utilised this data source, in which certain depressive symptoms, i.e. guilt feelings, impaired concentration, disturbed sleep, and delusions, were associated with a lower mortality risk in people with late-life depression [47]. The study also showed that severity of problems related to depressed mood on the HoNOS65+ subscale predicted

For peer review only - http://bmjopen.bmj.com/site/about/guidelines.xhtml

mortality only when patients with new-onset depression were considered [47]. This could be related to the sample being drawn from a secondary care provider for which a certain severity threshold needs to be met, and might explain why depression severity according to the HoNOS65+ subscale was not associated with an increased risk of dementia in the present study.

Strengths and limitations

Strengths of this study include the large, population-based sample in routine clinical care. The main limitations relate to the generalisability of the findings drawn from this selective cohort. To be captured in the study patients needed a secondary care diagnosis of depression, meaning these findings might not generalise to patients with milder depressive symptoms treated in primary care or those not readily seeking a depression diagnosis [48]. Crucially, the results drawn from examining the duration between depression and dementia diagnosis related to the time taken to receive a diagnosis of dementia in SLaM services, rather than the time taken to develop the diagnosable symptoms of dementia. This duration is influenced by potential barriers to timely diagnosis; service, provider, patient and care-giver factors contribute to delayed dementia diagnosis [49]. A review of the literature on missed and delayed diagnosis of dementia found symptom severity and degree of impairment are important predictors of diagnostic sensitivity [49]. Additionally, late-life depression confers an increased mortality risk, both in relation to suicide and when suicidality is accounted for [50], and the sub-cohort of patients who received both depression and dementia diagnoses in SLaM was selective to the 806 patients who survived long enough to receive a dementia diagnosis.

Of the 806 patients diagnosed with dementia, we found that 25% received an initial diagnosis of unspecified dementia and only 44% a diagnosis of Alzheimer's disease. This is lower than the expected incidence of Alzheimer's disease in two-thirds of dementia cases [51] and likely to reflect the naturalistic nature of the data, whereby the specific subtype is often recorded as unspecified until further investigations are conducted or more specific symptoms emerge. Subsequently the recording of specific subtypes increases if the whole record is considered [52 53].

For peer review only

Medication prescription was ascertained either within the 2 years before or after the index date of first depression diagnosis through natural language processing, but the time-dependent nature of prescribing could not be accounted for. Further, the output of natural language processing depends on the accuracy and quality of data entry, which varies by individual clinician and is compromised through the use of jargon, idiosyncratic abbreviations or misspellings [17]. Although it has been shown that precision and recall are relatively high for the medication natural language processing application [17], there remains a risk under- or overestimating the true prescribing prevalence, especially as adherence to medication cannot be established [54]. Lastly, although we incorporated a wide range of potential confounders in our analysis, causal inferences cannot be readily be drawn in an observational study of this nature.

Conclusions

The aim of our study was to highlight clinical markers of increased risk and accelerated progression to dementia in late-life depression. A knowledge of the indicators of the preclinical phase of dementia may be crucial to aid advancement in the identification, and consequently the management, of the disease at the earliest possible stage. As patients at the earliest stage of dementia are most likely to benefit from intervention to delay progression, future efforts to improve the detection of dementia should focus on recognizing subtle, early manifestations of disease. By examining the temporal relationships between characteristics within late-life depression and progression to dementia, in the context of South London secondary mental health service users, we have highlighted features that may indicate higher risk of and sooner progression to dementia. In older patients presenting with new-onset depression and/or more problematic cognitive symptoms, clinicians should suspect a diagnosis of dementia. It may be beneficial to closely monitor these patients for signs of cognitive deterioration suggestive of dementia, to facilitate the earliest possible detection and diagnosis. These patients might also form an interesting group for which novel pharmacological or psychosocial interventions could be developed.

For peer review only

Author Contributions:

GeP, CM, RS and DA conceived the study. GeP, CM and GaP performed the analysis and interpreted the data. GeP and CM wrote the manuscript. NK, MS, DA, RS provided expertise and feedback. All authors critically revised the manuscript and approved the final version of the study.

Data Availability Statement:

Deidentified patient data was obtained via the Clinical Record Interactive Search system (CRIS) at the National Institute for Health Research (NIHR) Biomedical Research Centre (BRC) at South London and Maudsley NHS Foundation Trust and the Institute of Psychiatry, Psychology & Neuroscience at King's College London. The data accessed by CRIS remain within an NHS firewall and governance is provided by a patient-led oversight committee. There are no additional unpublished data available.

For peer review only

References:

1. Byers AL, Yaffe K. Depression and risk of developing dementia. *Nat Rev Neurol* 2011;7(6):323-31. doi: 10.1038/nrneurol.2011.60 [published Online First: 2011/05/03]
2. Heo M, Murphy CF, Fontaine KR, et al. Population projection of US adults with lifetime experience of depressive disorder by age and sex from year 2005 to 2050. *Int J Geriatr Psychiatry* 2008;23(12):1266-70. doi: 10.1002/gps.2061 [published Online First: 2008/05/27]
3. Cherbuin N, Kim S, Anstey KJ. Dementia risk estimates associated with measures of depression: a systematic review and meta-analysis. *BMJ Open* 2015;5(12):e008853. doi: 10.1136/bmjopen-2015-008853 [published Online First: 2015/12/21]
4. Ownby RL, Crocco E, Acevedo A, et al. Depression and risk for Alzheimer disease: systematic review, meta-analysis, and metaregression analysis. *Archives of general psychiatry* 2006;63(5):530-8. doi: 10.1001/archpsyc.63.5.530 [published Online First: 2006/05/03]
5. Diniz BS, Butters MA, Albert SM, et al. Late-life depression and risk of vascular dementia and Alzheimer's disease: systematic review and meta-analysis of community-based cohort studies. *Br J Psychiatry* 2013;202(5):329-35. doi: 10.1192/bjp.bp.112.118307
6. Livingston G, Sommerlad A, Orgeta V, et al. Dementia prevention, intervention, and care. *Lancet* 2017;390(10113):2673-734. doi: 10.1016/S0140-6736(17)31363-6 [published Online First: 2017/07/20]
7. Kaup AR, Byers AL, Falvey C, et al. Trajectories of Depressive Symptoms in Older Adults and Risk of Dementia. *JAMA Psychiatry* 2016;73(5):525-31. doi: 10.1001/jamapsychiatry.2016.0004
8. Mirza SS, Wolters FJ, Swanson SA, et al. 10-year trajectories of depressive symptoms and risk of dementia: a population-based study. *Lancet Psychiatry* 2016;3(7):628-35. doi: 10.1016/S2215-0366(16)00097-3 [published Online First: 2016/04/29]
9. Singh-Manoux A, Dugravot A, Fournier A, et al. Trajectories of Depressive Symptoms Before Diagnosis of Dementia: A 28-Year Follow-up Study. *JAMA Psychiatry* 2017;74(7):712-18. doi: 10.1001/jamapsychiatry.2017.0660
10. Almeida OP, Hankey GJ, Yeap BB, et al. Depression as a modifiable factor to decrease the risk of dementia. *Transl Psychiatry* 2017;7(5):e1117. doi: 10.1038/tp.2017.90 [published Online First: 2017/05/02]
11. Mirza SS, de Bruijn RF, Direk N, et al. Depressive symptoms predict incident dementia during short- but not long-term follow-up period. *Alzheimers Dement* 2014;10(5 Suppl):S323-S29.e1. doi: 10.1016/j.jalz.2013.10.006 [published Online First: 2014/02/12]
12. Ismail Z, Smith EE, Geda Y, et al. Neuropsychiatric symptoms as early manifestations of emergent dementia: Provisional diagnostic criteria for mild behavioral impairment. *Alzheimers Dement* 2016;12(2):195-202. doi: 10.1016/j.jalz.2015.05.017 [published Online First: 2015/06/18]
13. Jorm AF. History of depression as a risk factor for dementia: an updated review. *Aust N Z J Psychiatry* 2001;35(6):776-81. doi: 10.1046/j.1440-1614.2001.00967.x
14. Donovan NJ, Locascio JJ, Marshall GA, et al. Longitudinal Association of Amyloid Beta and Anxious-Depressive Symptoms in Cognitively Normal Older

For peer review only

Adults. *Am J Psychiatry* 2018;175(6):530-37. doi: 10.1176/appi.ajp.2017.17040442 [published Online First: 2018/01/12]
15. Panza F, Frisardi V, Capurso C, et al. Late-life depression, mild cognitive impairment, and dementia: possible continuum? *Am J Geriatr Psychiatry* 2010;18(2):98-116. doi: 10.1097/JGP.0b013e3181b0fa13
16. Fernandes AC, Cloete D, Broadbent MT, et al. Development and evaluation of a de-identification procedure for a case register sourced from mental health electronic records. *BMC medical informatics and decision making* 2013;13:71. doi: 10.1186/1472-6947-13-71 [published Online First: 2013/07/12]
17. Perera G, Broadbent M, Callard F, et al. Cohort profile of the South London and Maudsley NHS Foundation Trust Biomedical Research Centre (SLaM BRC) Case Register: current status and recent enhancement of an Electronic Mental Health Record-derived data resource. *BMJ Open* 2016;6(3):e008721. doi: 10.1136/bmjopen-2015-008721
18. Cunningham H, Tablan V, Roberts A, et al. Getting more out of biomedical documents with GATE's full lifecycle open source text analytics. *PLoS computational biology* 2013;9(2):e1002854.
19. World Health O. International statistical classification of diseases and related health problems: World Health Organization 2004.
20. Uk GOV. English indices of deprivation 2010: Accessed, 2016.
21. Burns A, Beevor A, Lelliott P, et al. Health of the Nation Outcome Scales for elderly people (HoNOS 65+). *Br J Psychiatry* 1999;174:424-7.
22. StataCorp LP. Stata: release 13-statistical software. *College Station, TX* 2013
23. Oudshoorn CGM BS, Rijckevorsel JLA. Flexible multiple imputation by chained equations of the AVO-95 survey. *TNO Prevention and Health Leiden* 1999
24. DB R. Multiple imputation for nonresponse in surveys. *Vol 81: John Wiley & Sons* 2004
25. Stewart R, Soremekun M, Perera G, et al. The South London and Maudsley NHS Foundation Trust Biomedical Research Centre (SLAM BRC) case register: development and descriptive data. *BMC Psychiatry* 2009;9:51. doi: 10.1186/1471-244X-9-51 [published Online First: 2009/08/12]
26. Dotson VM, Beydoun MA, Zonderman AB. Recurrent depressive symptoms and the incidence of dementia and mild cognitive impairment. *Neurology* 2010;75(1):27-34. doi: 10.1212/WNL.0b013e3181e62124
27. Barnes DE, Yaffe K, Byers AL, et al. Midlife vs late-life depressive symptoms and risk of dementia: differential effects for Alzheimer disease and vascular dementia. *Archives of general psychiatry* 2012;69(5):493-8. doi: 10.1001/archgenpsychiatry.2011.1481
28. Wetherell JL, Gatz M, Johansson B, et al. History of depression and other psychiatric illness as risk factors for Alzheimer disease in a twin sample. *Alzheimer Dis Assoc Disord* 1999;13(1):47-52.
29. Gallagher D, Kiss A, Lanctot K, et al. Depression and Risk of Alzheimer Dementia: A Longitudinal Analysis to Determine Predictors of Increased Risk among Older Adults with Depression. *Am J Geriatr Psychiatry* 2018;26(8):819-27. doi: 10.1016/j.jagp.2018.05.002 [published Online First: 2018/05/09]
30. Heser K, Bleckwenn M, Wiese B, et al. Late-Life Depressive Symptoms and Lifetime History of Major Depression: Cognitive Deficits are Largely Due to Incipient Dementia rather than Depression. *J Alzheimers Dis* 2016;54(1):185-99. doi: 10.3233/JAD-160209

For peer review only

31. Amieva H, Le Goff M, Millet X, et al. Prodromal Alzheimer's disease: successive emergence of the clinical symptoms. *Ann Neurol* 2008;64(5):492-8. doi: 10.1002/ana.21509
32. Wang YC, Tai PA, Poly TN, et al. Increased Risk of Dementia in Patients with Antidepressants: A Meta-Analysis of Observational Studies. *Behav Neurol* 2018;2018:5315098. doi: 10.1155/2018/5315098 [published Online First: 2018/08/21]
33. Kodesh A, Sandin S, Reichenberg A, et al. Exposure to Antidepressant Medication and the Risk of Incident Dementia. *Am J Geriatr Psychiatry* 2019;27(11):1177-88. doi: 10.1016/j.jagp.2019.05.019 [published Online First: 2019/06/27]
34. Modrego PJ, Ferrández J. Depression in patients with mild cognitive impairment increases the risk of developing dementia of Alzheimer type: a prospective cohort study. *Arch Neurol* 2004;61(8):1290-3. doi: 10.1001/archneur.61.8.1290
35. Tedeschini E, Levkovitz Y, Iovieno N, et al. Efficacy of antidepressants for late-life depression: a meta-analysis and meta-regression of placebo-controlled randomized trials. *J Clin Psychiatry* 2011;72(12):1660-8. doi: 10.4088/JCP.10r06531 [published Online First: 2012/01/17]
36. Tham A, Jonsson U, Andersson G, et al. Efficacy and tolerability of antidepressants in people aged 65 years or older with major depressive disorder - A systematic review and a meta-analysis. *Journal of affective disorders* 2016;205:1-12. doi: 10.1016/j.jad.2016.06.013 [published Online First: 2016/07/09]
37. Bishara D, Harwood D, Sauer J, et al. Anticholinergic effect on cognition (AEC) of drugs commonly used in older people. *Int J Geriatr Psychiatry* 2017;32(6):650-56. doi: 10.1002/gps.4507
38. Nebes RD, Pollock BG, Houck PR, et al. Persistence of cognitive impairment in geriatric patients following antidepressant treatment: a randomized, double-blind clinical trial with nortriptyline and paroxetine. *J Psychiatr Res* 2003;37(2):99-108.
39. Bhalla RK, Butters MA, Mulsant BH, et al. Persistence of neuropsychologic deficits in the remitted state of late-life depression. *Am J Geriatr Psychiatry* 2006;14(5):419-27. doi: 10.1097/01.JGP.0000203130.45421.69
40. Butters MA, Becker JT, Nebes RD, et al. Changes in cognitive functioning following treatment of late-life depression. *Am J Psychiatry* 2000;157(12):1949-54. doi: 10.1176/appi.ajp.157.12.1949
41. Devanand DP, Pelton GH, Marston K, et al. Sertraline treatment of elderly patients with depression and cognitive impairment. *Int J Geriatr Psychiatry* 2003;18(2):123-30. doi: 10.1002/gps.802
42. Reid LD, Avens FE, Walf AA. Cognitive behavioral therapy (CBT) for preventing Alzheimer's disease. *Behav Brain Res* 2017;334:163-77. doi: 10.1016/j.bbr.2017.07.024 [published Online First: 2017/07/27]
43. Sommerlad A, Sabia S, Singh-Manoux A, et al. Association of social contact with dementia and cognition: 28-year follow-up of the Whitehall II cohort study. *PLoS Med* 2019;16(8):e1002862. doi: 10.1371/journal.pmed.1002862 [published Online First: 2019/08/03]
44. Cassidy-Eagle E, Siebern A, Unti L, et al. Neuropsychological Functioning in Older Adults with Mild Cognitive Impairment and Insomnia Randomized to

For peer review only

CBT-I or Control Group. *Clin Gerontol* 2018;41(2):136-44. doi: 10.1080/07317115.2017.1384777 [published Online First: 2017/12/09]
45. Dafsari FS, Bewernick B, Biewer M, et al. Cognitive behavioural therapy for the treatment of late life depression: study protocol of a multicentre, randomized, observer-blinded, controlled trial (CBTlate). *BMC Psychiatry* 2019;19(1):423. doi: 10.1186/s12888-019-2412-0 [published Online First: 2019/12/29]
46. Colak E, Ozen H, Emir B, et al. Pairwise Multiple Comparison Adjustment Procedure for Survival Functions with Right-Censored Data. *Comput Math Methods Med* 2017;2017:9270450. doi: 10.1155/2017/9270450 [published Online First: 2017/11/11]
47. Cai W, Mueller C, Shetty H, et al. Predictors of mortality in people with late-life depression: A retrospective cohort study. *Journal of affective disorders* 2020
48. Winblad B, Amouyel P, Andrieu S, et al. Defeating Alzheimer's disease and other dementias: a priority for European science and society. *Lancet Neurol* 2016;15(5):455-532. doi: 10.1016/S1474-4422(16)00062-4
49. Bradford A, Kunik ME, Schulz P, et al. Missed and delayed diagnosis of dementia in primary care: prevalence and contributing factors. *Alzheimer Dis Assoc Disord* 2009;23(4):306-14. doi: 10.1097/WAD.0b013e3181a6bebc
50. Hall CA, Reynolds-Iii CF. Late-life depression in the primary care setting: challenges, collaborative care, and prevention. *Maturitas* 2014;79(2):147-52. doi: 10.1016/j.maturitas.2014.05.026 [published Online First: 2014/07/06]
51. Corriveau RA, Koroshetz WJ, Gladman JT, et al. Alzheimer's Disease-Related Dementias Summit 2016: National research priorities. *Neurology* 2017;89(23):2381-91. doi: 10.1212/WNL.0000000000004717 [published Online First: 2017/11/10]
52. Mueller C, Perera G, Rajkumar A, et al. Hospitalization in people with dementia with Lewy bodies: Frequency, duration, and cost implications. *Alzheimer's & Dementia: Diagnosis, Assessment & Disease Monitoring* 2018;10:143-52.
53. Mueller C, Molokhia M, Perera G, et al. Polypharmacy in people with dementia: Associations with adverse health outcomes. *Experimental Gerontology* 2018 [published Online First: 13 Feb 2018]
54. Cai W, Mueller C, Shetty H, et al. Predictors of cerebrovascular event reoccurrence in patients with depression: a retrospective cohort study. *BMJ Open* 2020;10(1):e031927. doi: 10.1136/bmjopen-2019-031927 [published Online First: 2020/01/10]

For peer review only

Figure legends/captions:

Figure 1
Title: Flowchart showing cohort generation and outcomes.

Figure 2
Title: Plot of hazard ratios for risk of dementia diagnosis in a multivariate Cox regression model adjusted for all covariates in 3,659 patients with late-life depression, of whom 806 developed dementia.
Legend:
Coloured points indicate p<.05
[a] at or closest to the time of depression diagnosis.
[b] per 10 unit increase in IMD score.
[c] Recurrent depressive disorder diagnosis or previous diagnosis of depression.
[d] Health of the Nation Outcome Scale 65+ (HoNOS65+) subscale scores 0-4 (0=least severe, 4 = most severe status). Values represent frequencies of patients scored as experiencing problems (score 2-4) in that domain.

Figure 3
Title: Impact of significant risk factors on time to dementia diagnosis in 806 patients with late-life depression who were subsequently diagnosed with dementia during follow-up.
Legend:
[a] at or closest to the time of depression diagnosis.
[b] Health of the Nation Outcome Scale 65+ (HoNOS65+) subscale scores 0-4 (0=least severe, 4 = most severe status). Values represent frequencies of patients scored as experiencing problems (score 2-4) in that domain.
[c] Recurrent depressive disorder diagnosis or previous diagnosis of depression.

For peer review only

Figure 1. Flowchart showing cohort generation and outcomes.

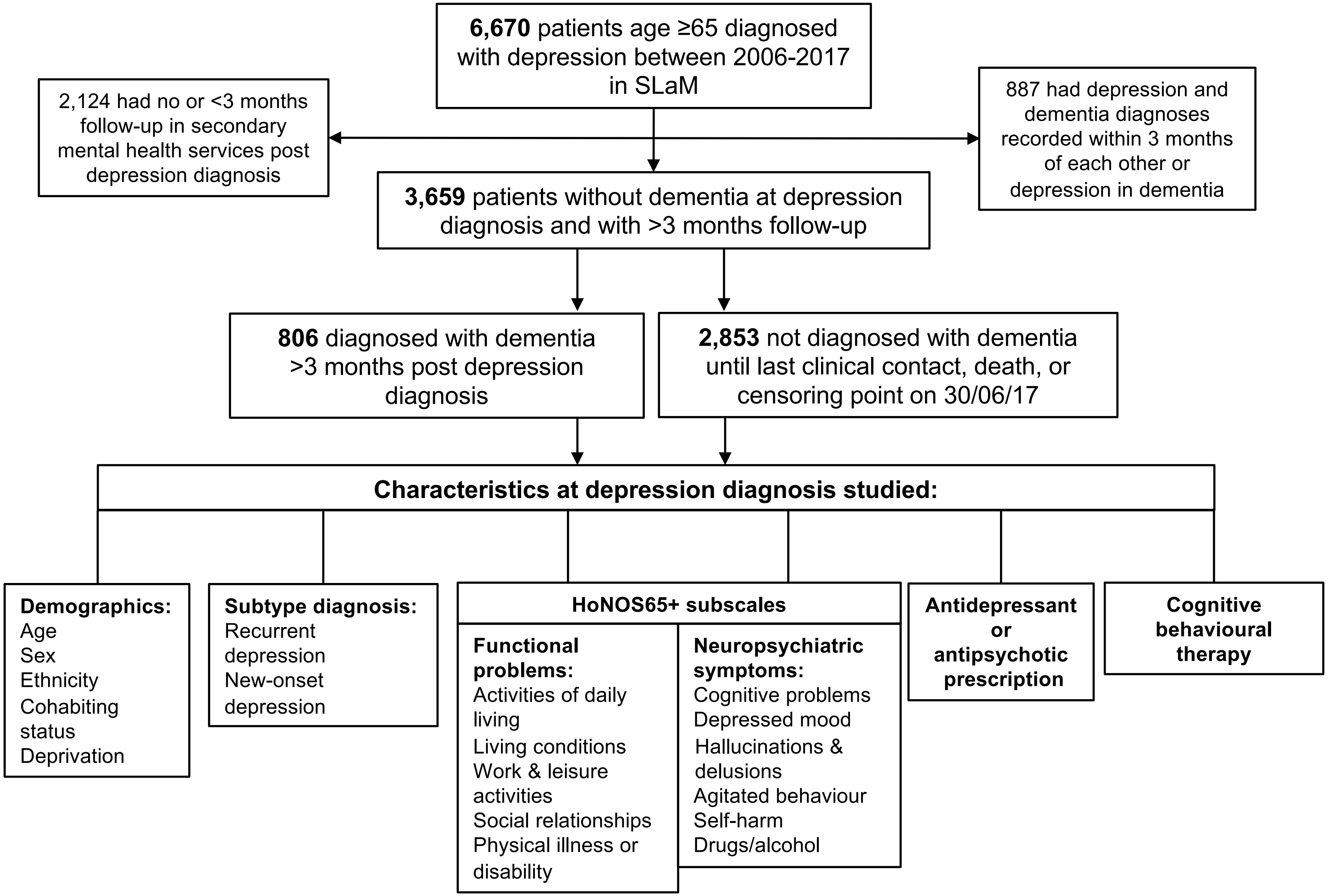

Figure 2. Plot of hazard ratios for risk of dementia diagnosis in a multivariate Cox regression model adjusted for all covariates in 3,659 patients with late-life depression, of whom 806 developed dementia.

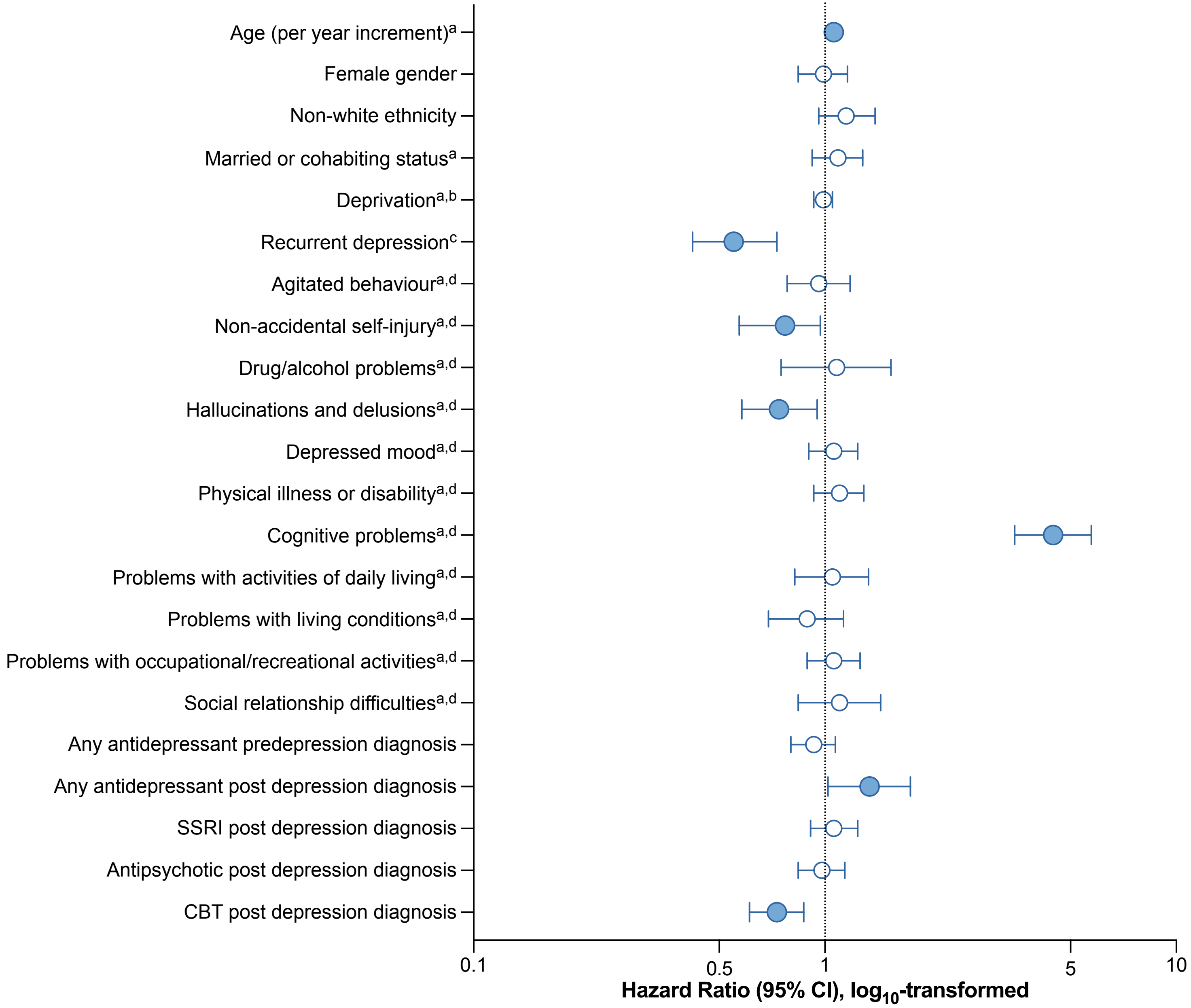

Figure 3. Impact of significant risk factors on time to dementia diagnosis in 806 patients with late-life depression who were subsequently diagnosed with dementia during follow-up.

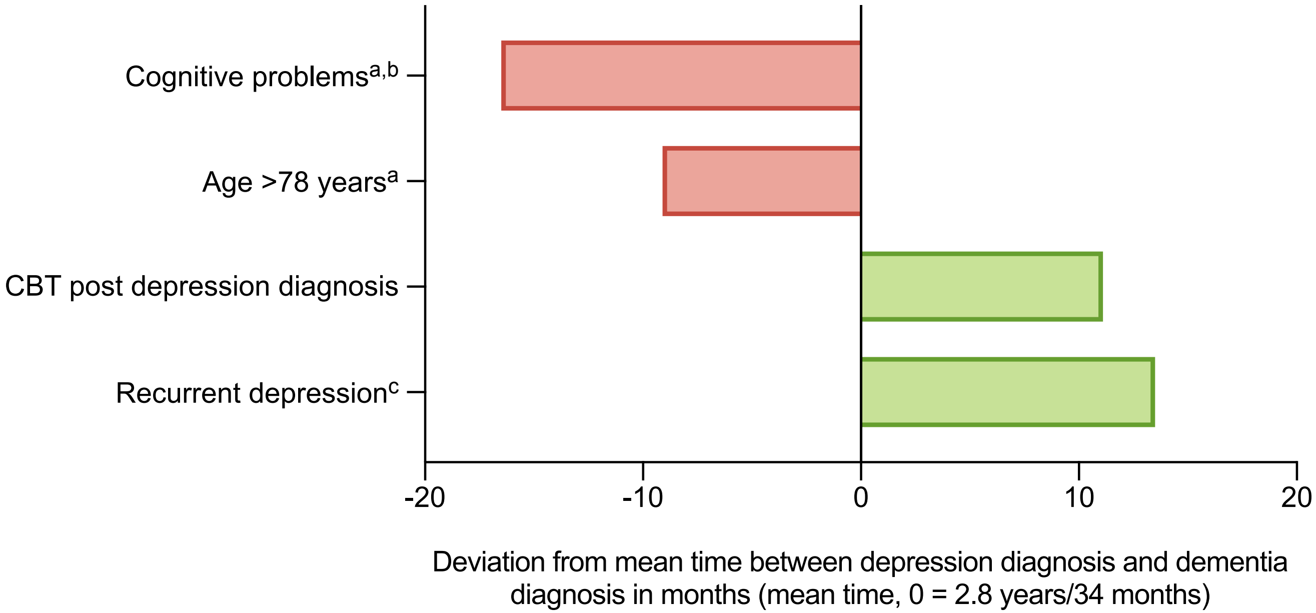

BMJ Open

Clinical factors associated with progression to dementia in people with late-life depression: a cohort study of patients in secondary care

Journal:	*BMJ Open*
Manuscript ID	bmjopen-2019-035147.R2
Article Type:	Original research
Date Submitted by the Author:	06-Apr-2020
Complete List of Authors:	Peakman, Georgia; IoPPN, Old Age Psychiatry;<br>Karunatilake, Nishshanka; South London and Maudsley NHS Foundation Trust<br>Seynaeve, Mathieu; South London and Maudsley NHS Foundation Trust; King's College London Institute of Psychiatry<br>Perera, Gayan; Institute of Psychiatry, Psychological Medicine<br>Aarsland, Dag; Stavanger University Hospital; King's College London Institute of Psychiatry, Department of Old Age Psychiatry<br>Stewart, Robert; King's College London, Institute of Psychiatry; South London and Maudsley NHS Foundation Trust<br>Mueller, Christoph; King's College London Institute of Psychiatry, Department of Old Age Psychiatry; South London and Maudsley NHS Foundation Trust
**Primary Subject Heading**:	Mental health
Secondary Subject Heading:	Geriatric medicine, Neurology
Keywords:	Depression & mood disorders < PSYCHIATRY, Dementia < NEUROLOGY, GERIATRIC MEDICINE, MENTAL HEALTH

SCHOLARONE™
Manuscripts

I, the Submitting Author has the right to grant and does grant on behalf of all authors of the Work (as defined in the below author licence), an exclusive licence and/or a non-exclusive licence for contributions from authors who are: i) UK Crown employees; ii) where BMJ has agreed a CC-BY licence shall apply, and/or iii) in accordance with the terms applicable for US Federal Government officers or employees acting as part of their official duties; on a worldwide, perpetual, irrevocable, royalty-free basis to BMJ Publishing Group Ltd ("BMJ") its licensees and where the relevant Journal is co-owned by BMJ to the co-owners of the Journal, to publish the Work in this journal and any other BMJ products and to exploit all rights, as set out in our licence.

The Submitting Author accepts and understands that any supply made under these terms is made by BMJ to the Submitting Author unless you are acting as an employee on behalf of your employer or a postgraduate student of an affiliated institution which is paying any applicable article publishing charge ("APC") for Open Access articles. Where the Submitting Author wishes to make the Work available on an Open Access basis (and intends to pay the relevant APC), the terms of reuse of such Open Access shall be governed by a Creative Commons licence – details of these licences and which Creative Commons licence will apply to this Work are set out in our licence referred to above.

Other than as permitted in any relevant BMJ Author's Self Archiving Policies, I confirm this Work has not been accepted for publication elsewhere, is not being considered for publication elsewhere and does not duplicate material already published. I confirm all authors consent to publication of this Work and authorise the granting of this licence.

For peer review only

Clinical factors associated with progression to dementia in people with late-life depression: a cohort study of patients in secondary care

Georgia Peakman[1], Nishshanka Karunatilake[2], Mathieu Seynaeve[1,2], Gayan Perera[1], Dag Aarsland[1,3], Robert Stewart[1,2], Christoph Mueller[1,2]

[1]King's College London, Institute of Psychiatry, Psychology and Neuroscience, London, UK

[2]South London and Maudsley NHS Foundation Trust, London, UK

[3]Centre for Age-Related Medicine, Stavanger University Hospital, Stavanger, Norway

Corresponding author: Georgia Peakman; King's College London, Institute of Psychiatry, Psychology and Neuroscience (IoPPN), De Crespigny Park, London, SE5 8AF, United Kingdom; email: georgia.peakman.18@ucl.ac.uk; phone: +44 207 848 0626.

For peer review only

Declarations of interest:

RS has received research funding from Roche, Pfizer, Janssen, Lundbeck and In-Silico-Bioscience. DA has received research support and/or honoraria from Astra-Zeneca, H. Lundbeck, Novartis Pharmaceuticals and GE Health, and serves as paid consultant for H. Lundbeck and Axovant. All other authors declare no conflict of interest.

Funding:

GeP received undergraduate bursaries from the Alzheimer's Society and the Wellcome Trust to conduct this project. CM, GaP, DA and RS receive salary support from the National Institute for Health Research (NIHR) Biomedical Research Centre at South London and Maudsley NHS Foundation Trust and King's College London, and RS is a NIHR Senior Investigator. The views expressed are those of the author(s) and not necessarily those of the NHS, the NIHR or the Department of Health.

Word count: 4,204

Tables: 3

Figures: 3

For peer review only

Clinical factors associated with progression to dementia in people with late-life depression: a cohort study of patients in secondary care.

Objectives: Depression can be a prodromal feature or a risk factor for dementia. We aimed to investigate which clinical factors in patients with late-life depression are associated with a higher risk of developing dementia and a more rapid conversion.

Design: Retrospective cohort study.

Setting: South London and Maudsley NHS Foundation Trust (SLaM) secondary mental healthcare services.

Participants: The SLaM Clinical Record Interactive Search (CRIS) was used to retrieve anonymised data on 3,659 patients aged 65 years or older who had received a diagnosis of depression in mental health services and had been followed-up for at least 3 months.

Outcome measures: Predictors of development of incident dementia were investigated, including demographic factors, health status rated on the Health of the National Outcome scale for older people (HoNOS65+), depression recurrence, and treatments including psychotropic drugs and cognitive behavioural therapy (CBT).

Results: In total, 806 (22.0%) patients developed dementia over a mean follow-up time of 2.7 years. Significant predictors of receiving a dementia diagnosis in fully adjusted models and after accounting for multiple comparisons were: older age (adjusted hazard ratio HR=1.04, 95% CI: 1.03-1.06 per year difference from sample mean) and the HoNOS65+ subscale measuring cognitive problems (HR=4.72, 95% CI: 3.67-6.06 for scores in the problematic range). Recurrent depressive disorder or past depression (HR=0.65, 95% CI: 0.55-0.77) and the receipt of CBT (HR=0.73 95% CI: 0.61-0.87) were associated with a lower dementia risk. Over time, hazards related to age increased and hazards related to cognitive problems decreased.

Conclusions: In older adults with depression, a higher risk of being subsequently diagnosed with dementia was predicted by higher age, new onset depression, severity of cognitive symptoms and not receiving CBT. Further exploration is needed to determine whether the latter risk factors are responsive to interventions.

Key words: depression, dementia, diagnosis, predictors, secondary care.

For peer review only

Article Summary

Strengths and limitations of this study

- This study used a large naturalistic sample of older patients receiving routine mental healthcare, with access to anonymised clinical data.
- The sample was limited to patients who had been diagnosed with depression, followed-up, and if applicable, diagnosed with dementia within the same secondary provider; it is not readily generalisable to cases of depression treated in other settings.
- Time between depression diagnosis and dementia diagnosis is additionally influenced by potential barriers to seeking a dementia diagnosis such as service, provider, patient and care-giver factors, which were not captured by this analysis.
- The influences of other comorbid long-term conditions and resultant polypharmacy were not fully considered, and causal inferences cannot be readily made from observational data.

For peer review only

Introduction

A link between depression and dementia is well established, and growing evidence indicates that the timing of depression is important in defining the nature of the relationship [1]. Late-life depression is projected to increase substantially by 2050 [2] and has consistently been associated with up to a two-fold increased risk for dementia [3-5], and it is now recognised as a key potentially modifiable risk factor for the condition [6]. However, causality is yet to be established; it remains unclear whether, and in which cases, late-life depression is an independent risk factor for dementia or a prodromal symptom.

Identification of the clinical characteristics of older adults with depression that are indicative of incipient dementia would facilitate accurate classification of individuals at high risk for early progression and subsequently, timelier treatment. The pattern and severity of depressive symptoms in late life has emerged as one important factor in determining the likelihood of progression to dementia. In two large, longitudinal cohort studies of older adults, those subjects characterised by trajectories of high and steadily increasing depressive symptoms over several years were significantly more likely to develop dementia [7, 8]. Having an episode of high depressive symptoms that remitted was not associated with greater dementia risk [8]. Similarly, recent data from the Whitehall II cohort study characterised those with late-life depression who later developed dementia as having a distinguishable trajectory of increasing depressive symptoms, emerging 11 years before dementia diagnosis [9]. Depressive symptoms during mid-life, even when recurrent, did not increase dementia risk in this cohort. These findings, in addition to other reports of graded associations with depression severity and raised dementia risk being contingent on the two being in closer temporal proximity [10, 11], lend support to the hypotheses that depressive symptoms during later life can be an early manifestation of dementia [12], or that the two conditions share common aetiological factors [13, 14]; potentially both are consequences of an underlying neurodegenerative process [15].

While it is well established that greater severity of depressive symptoms is closely associated with accelerated development of dementia, other associations relevant to

For peer review only

this relationship are less clear. Using naturalistic data from a cohort of secondary mental health service users, the present study examined associations between patient characteristics and interventions within late-life depression and the risk of progression to a diagnosis of dementia.

For peer review only

Methods

Source of data

Data for this study were obtained via the South London and Maudsley NHS Foundation Trust (SLaM), one of Europe's largest mental healthcare providers, serving 1.3 million residents in four south London boroughs (Croydon, Lambeth, Lewisham and Southwark), as well as delivering specialist national services. SLaM adopted fully electronic records across all of its services from 2006, having imported legacy systems which include a records system run for several years previously in dementia care. The Clinical Record Interactive Search (CRIS) application was developed in 2008 to provide access to de-identified structured and open-text data extracted from this electronic health records system, containing records on over 400,000 SLaM mental health service users [16, 17]. CRIS has been enhanced by a range of natural language processing ('text-mining') applications [18] which extract structured data from text fields, including applications to ascertain pharmacotherapy [17]. CRIS has research ethical approval as an anonymised database for secondary analysis (Oxford REC C 08/H0606/71+5).

Sample

Using CRIS, people aged 65 or older who received a diagnosis of depression from SLaM services within the 11-year period between 01/01/2006 to 30/03/2017 were identified according to the International Statistical Classification of Diseases and Related Health Problems 10th Revision (ICD-10) [19] criteria described below. Date of first depression diagnosis after the age of 65 years served as the index date. Patients were followed-up until the date of their first dementia diagnosis (incident dementia) given in the health record; or their last face-to-face contact with a professional from secondary mental health services (clinical event or appointment); or death; or a censoring point on 30/06/2017. The censored times therefore correspond to all events bar the occurrence of the patient's first diagnosis. Patients were excluded if they had a diagnosis of dementia recorded before or within 3 months of the index date, or if they had less than 3 months of contact with SLaM services after their depression diagnosis.

Measurements

For peer review only

Cases of depression and dementia according to ICD-10 codes were ascertained, both from diagnoses recorded in structured fields and in free text within clinical correspondence, through a natural language processing algorithm [18].

Late-life depression

Initial depression diagnoses (the index contact) were determined by the following diagnostic codes from ICD-10 [19] within a primary or secondary diagnosis: F32 (depressive episode), F32.2 (severe depressive episode without psychotic symptoms), F32.3 (severe depressive episode with psychotic symptoms), F33 (recurrent depressive disorder), F33.2 (recurrent depressive disorder, current episode severe without psychotic symptoms), F33.3 (recurrent depressive disorder, current episode severe with psychotic symptoms). Data were also obtained for any depression diagnoses (F32, F33) recorded before the index date.

Dementia

Dementia diagnoses were determined by the following diagnostic codes from ICD-10[19] within a primary or secondary diagnosis: F00 (dementia in Alzheimer's disease), F01 (vascular dementia), F02 (dementia in other diseases classified elsewhere), F03 (unspecified dementia).

Covariates

Data were also extracted on age (at depression diagnosis), gender, ethnicity, marital or cohabiting status, local authority, a neighbourhood-level deprivation score (Index of Multiple Deprivation, 2010) [20], psychotropic medication (classified into antipsychotic, antidepressant and selective serotonin reuptake inhibitor (SSRI) types) prescribed in the period 2 years prior to depression diagnosis and following diagnosis, and the receipt of cognitive behaviour therapy (CBT) after depression diagnosis. Age was centred in the regression models, meaning that incremental effects related to a one-year difference from the mean. The Health of the Nation Outcome Scales for older people [21] (HoNOS65+) is routinely administered in SLaM services, and the subscale scores available closest to the depression diagnosis extracted for this analysis were: agitated behaviour, non-accidental self-injury, drug/alcohol problems, hallucinations or delusions, depressed mood, physical illness or disability, cognitive problems, activities of daily living (ADLs), living conditions, occupational and recreational

For peer review only

activities, social relationships. HoNOS65+ items are rated from 0 (least severe status) to 4 (most severe status). For the purpose of this analysis scores of 0-1 were considered to indicate that the patient was not experiencing problems in the domain, and scores of 2-4 were considered to indicate experiencing severe problems as rated by the clinician.

Statistical Analysis

Baseline characteristics of the cohort grouped according to dementia development (those who were diagnosed with dementia within secondary mental health services and those who didn't have a dementia diagnosis at their last contact with services), were compared using chi-square tests for categorical variables, and independent samples t-tests or analysis of variance (ANOVA) for continuous variables.

Cox proportional hazards regression models were used to analyse associations between the predictor variables at depression diagnosis and hazard of receiving a diagnosis of dementia. Multivariate models were first adjusted for demographic factors; age, gender and deprivation (Model 1), then for the HoNOS65+ variables of cognitive problems and depressed mood (Model 2), and finally all variables were adjusted for in Model 3 to determine independent associations. All analyses were performed using Stata version 13.0 [22]. As 19% of the cohort had missing data on at least one covariate, we used the *mi* package in STATA to create 19 imputed datasets, generated by replacing missing values with simulated values assembled from potential covariates and outcome values [23]. Rubin's rules [24] were applied to combine coefficients in final analyses. We assessed the proportional-hazard assumptions for each variable on the basis of Schoenfeld residuals in the complete-case sample and included variables as time-varying if they were violated (both in the complete-case and imputed sample). We report results at both a significance level of $p<.05$ and a Bonferroni corrected $p<.002$ to account for multiple comparisons (22 variables examined). In a second step, we only considered those patients in the cohort who had a diagnosis of dementia recorded, to determine how the duration from depression to dementia diagnosis varied according to the predictors that had yielded significance in the above Cox regression models.

For peer review only

Patient and public involvement

A patient-led committee provides operational oversight of the CRIS resource and ensures that the proposed study has both scientific value as well as benefit for patients and carers [17, 25]. The authors had applied to the oversight committee with an outline of the proposed analysis, including data linkages, and the project was approved on 31/05/2017. There was no further patient and public involvement in this register-based study.

For peer review only

Results

Sample size and characteristics

During the 11-year study period, a total of 6,670 patients diagnosed with depression at age 65 or above were identified (Figure 1). Of these, 887 patients were excluded because they already had a co-morbid diagnosis of dementia at the time or within 3 months of the depression diagnosis. A further 2,124 patients were excluded because they were followed-up for less than 3 months after their depression diagnosis. The final cohort consisted of 3,659 patients with late-life depression, of which 806 (22.0%) received a diagnosis of dementia from mental health services during the follow-up period. Mean follow-up time until dementia diagnosis, last clinical face-to-face contact, census or death was 2.7 years (SD +/- 2.6 years); and this did not differ significantly between those who were or were not diagnosed with dementia (see Table 1).

Baseline characteristics of the late-life depression cohort as a whole and according to dementia development are presented in Table 1. Patients who were subsequently diagnosed with dementia were significantly older and more likely to be female, have cognitive problems and impairment of their activities of daily living (ADLs). They were less likely to have recurrent depression, non-accidental self-injury recorded, or problems related to their depressed mood or social relationships. In terms of pharmacotherapy, those who were diagnosed with dementia were more likely to be prescribed any antidepressant, a selective serotonin reuptake inhibitor (SSRI), or an antipsychotic after depression diagnosis, but were less likely to have received cognitive behavioural therapy (CBT).

For peer review only

Table 1: Baseline characteristics of the late-life depression cohort according to whether a dementia diagnosis was established.

**Baseline characteristics**	**Whole cohort**	**Dementia diagnosis >3 months post depression diagnosis**	**No dementia diagnosis during follow-up**	***p*-value[a]**
No. of patients	3,659	806	2,853	
Mean age (SD)[b]	76.0 (7.7)	78.3 (7.2)	75.3 (7.7)	<.001
Mean time in years from depression diagnosis to census (dementia/death/last clinical contact) (SD)	2.7 (2.6)	2.8 (2.4)	2.7 (2.6)	.365
Female (%)	64.1	68.7	62.7	.002
Non-white ethnicity (%)	17.6	19.4	17.1	.121
Married or cohabiting status (%)[b]	32.1	29.6	32.8	.091
Mean index of deprivation (SD)[b]	27.0 (11.5)	26.7 (11.3)	27.0 (11.5)	.487
F33 recurrent depressive disorder diagnosis (%)	28.5	23.2	30.0	<.001
**HoNOS65+ mental and physical health problems (%)[b,c]**				
Agitated behaviour	15.9	16.2	15.8	.775
Non-accidental self-injury	12.4	7.9	13.8	<.001
Drug/alcohol problems	5.6	4.4	6.0	.100
Hallucinations or delusions	11.3	10.2	11.7	.232
Depressed mood	67.4	61.8	69.1	<.001
Physical illness or disability	60.8	61.1	60.8	.846
Cognitive problems	19.1	35.7	14.1	<.001
**HoNOS65+ functional problems (%)[b,c]**				
Activities of daily living	44.1	47.2	43.1	.042
Living conditions	13.1	11.7	13.6	.168
Occupational and recreational activities	36.0	35.0	36.3	.526
Social relationships	25.2	21.9	26.2	.015
**Psychotropic use: in 2 years before depression diagnosis**				
Antidepressant (%)	56.1	55.6	56.3	.720
**Psychotropic use: after depression diagnosis**				
Antidepressant (%)	86.5	91.2	85.1	<.001
SSRI (%)	55.2	61.3	53.4	<.001
Antipsychotic (%)	34.4	38.5	33.2	.006
**Cognitive behavioural therapy**				
Received CBT post-depression diagnosis (%)	26.8	22.2	28.1	.001

[a] χ^2 test, analysis of variance or independent samples t-test
[b] at or closest to the time of depression diagnosis
[c] Health of the Nation Outcome Scale 65+ (HoNOS65+) subscale scores 0-4 (0 = least severe, 4 = most severe status). Values represent frequencies of patients scored as experiencing problems (score 2-4) in that domain.

For peer review only

Predictors of receiving a dementia diagnosis in secondary care services

Table 2 presents the findings from a series of multivariate Cox proportional hazards models examining associations between the predictors in late-life depression and risk of dementia diagnosis. Analyses were first adjusted for the demographic factors of age, gender and deprivation (Model 1). In this model, when considering $p<.002$ as significant to account for multiple comparisons, older age (as per year differing from the sample mean of 76.0 years) and non-white ethnicity, as well as the HoNOS65+ subscale scores recording problems with physical illness, cognition and ADLs were each significantly associated with a higher risk of developing dementia, while recurrent depression and treatment with CBT were associated with lower dementia risk. Both the protective effect of having recurrent depression and the hazardous effect of cognitive problems lessened over time. In Model 2 and the fully adjusted Model 3 (presented in Figure 2), at a significance level of $p<.002$, older age and cognitive problems remained associated with progression to dementia, and recurrent depression and treatment with CBT both remained associated with lower dementia risk. In these two models, the hazard associated with age increased and the hazard related to cognitive problems decreased with time. When applying a significance level of $p<.05$ (not corrected for multiple comparisons), presenting with non-accidental self-injury or hallucinations and/or delusions at depression diagnosis were associated with a lower risk of dementia, while those prescribed any antidepressant had a 33% increased dementia risk. The hazard associated with hallucinations and/or delusions increased with time.

Alternatively adding age as a squared term to the analyses did not alter the predictors yielding significance in the fully adjusted model (Model 3), with the exception of non-accidental self-injury ($p=0.076$) and antidepressant prescription after index date ($p=0.056$) which both attenuated to a trend. Similar results were found in the complete case sample which included 2,952 patients, of whom 717 developed dementia (see Supplementary Table 1).

For peer review only

Table 2: Results from a series of multivariate Cox proportional hazards models analysing associations between predictor variables and risk of dementia diagnosis (adjusted hazard ratio (95% CI)) in 3,659 patients with late-life depression, of whom 806 were subsequently diagnosed with dementia during study follow-up.

Predictor variables	**Model 1** Adjusted for age, gender, deprivation	**Model 2** Model 1 + depressed mood[b] + cognitive problems[b]	**Model 3** Adjusted for all predictor variables
**Demographics[a]**			
Centred age (per year difference from mean)	1.07 (1.06-1.08)**	1.05 (1.04-1.06)**	1.04 (1.03-1.06)**
*Centred age * time[c]*		*1.01 (1.00-1.01)**	*1.01 (1.00-1.01)**
Female gender	0.95 (0.82-1.11)	0.97 (0.83-1.12)	0.99 (0.84-1.16)
Non-white ethnicity	1.36 (1.13-1.62)**	1.22 (1.02-1.46)*	1.14 (0.95-1.37)
Married or cohabiting status	1.07 (0.91-1.25)	1.10 (0.94-1.29)	1.09 (0.92-1.28)
Deprivation (per 10 unit increase in IMD score)	1.01 (0.95-1.08)	0.99 (0.94-1.06)	0.99 (0.93-1.06)
Recurrent depressive disorder diagnosis or previous diagnosis of depression	0.47 (0.36-0.62)**	0.65 (0.55-0.77)**	0.65 (0.55-0.77)**
*Recurrent depression * time[c]*	*1.09 (1.02-1.16)**		
**HoNOS65+ mental and physical health problems[a,b]**			
Agitated behaviour	1.09 (0.90-1.33)	0.87 (0.71-1.06)	0.95 (0.77-1.17)
Non-accidental self-injury	0.69 (0.53-0.88)*	0.68 (0.52-0.88)*	0.76 (0.59-0.99)*
Drug/alcohol problems	1.03 (0.72-1.48)	0.97 (0.68-1.40)	1.09 (0.77-1.55)
Hallucinations and delusions	0.88 (0.70-1.10)	0.57 (0.39-0.83)*	0.57 (0.38-0.84)*
*Hallucinations and delusions * time[c]*		*1.08 (0.99-1.18)*	*1.10 (1.00-1.21)**
Depressed mood	1.11 (0.96-1.28)	1.02 (0.88-1.18)	1.05 (0.90-1.24)
Physical illness or disability	1.33 (1.15-1.54)**	1.18 (1.02-1.38)*	0.97 (0.76-1.23)
*Physical illness or disability * time[c]*			*1.05 (0.99-1.12)*
Cognitive problems	4.89 (3.84-6.21)**	4.96 (3.89-6.32)**	4.72 (3.67-6.06)**
*Cognitive problems * time[c]*	*0.84 (0.78-0.91)***	*0.84 (0.78-0.91)***	*0.84 (0.77-0.91)***
**HoNOS65+ functional problems[a,b]**			
Activities of daily living	1.43(1.24-1.65)**	1.12 (0.96-1.31)	1.06 (0.90-1.26)
Living conditions	1.04 (0.83-1.31)	0.88 (0.69-1.12)	0.87 (0.68-1.12)
Occupational/recreational activities	1.13 (0.97-1.32)	1.02 (0.87-1.19)	1.06 (0.89-1.26)
Social relationships	1.10 (0.85-1.42)	1.03 (0.79-1.33)	1.13 (0.86-1.48)
*Social relationships * time[c]*	*0.92 (0.85-1.42)**	*0.93 (0.87-1.00)**	*0.92 (0.85-0.99)**
**Psychotropic use: in 2 years before depression diagnosis**			
Antidepressant	0.87 (0.76-1.00)*	0.90 (0.78-1.04)	0.93 (0.80-1.08)
**Psychotropic use: after depression diagnosis**			
Any antidepressant	1.21 (0.95-1.55)	1.27 (0.99-1.61)	1.32 (1.01-1.74)*
SSRI	1.12 (0.97-1.29)	1.11 (0.96-1.28)	1.07 (0.91-1.25)
Antipsychotic	0.88 (0.76-1.02)	0.85 (0.73-0.99)*	0.98 (0.84-1.14)
**Cognitive behavioural therapy**			
Received CBT post-depression diagnosis	0.62 (0.52-0.73)**	0.69 (0.58-0.82)**	0.73 (0.61-0.87)**

*$p<.05$
**$p<.002$ (Bonferroni corrected)
[a] at or closest to the time of depression diagnosis
[b] Health of the Nation Outcome Scale 65+ (HoNOS65+) subscale scores 0-4 (0 = least severe, 4 = most severe status). Values represent frequencies of patients scored as experiencing problems (score 2-4) in that domain.
[c] time-variable-interactions describing how the hazard in the variable reported above changes per year.

For peer review only

Characteristics of 806 patients who received a dementia diagnosis

In those who received a dementia diagnosis, the average time from depression diagnosis to dementia diagnosis was 2.8 years (SD +/- 2.4). Of this group, the first recorded dementia subtype was Alzheimer's disease for 353 (43.8%), vascular dementia for 199 (24.7%), dementia in in other diseases for 51 (6.3%), and unspecified dementia for 203 (25.2%) patients.

Figure 3 presents variations in the mean time between depression and dementia diagnosis, according to the factors that were significantly associated with dementia risk in the fully adjusted model (Model 3) after accounting for multiple comparisons. An age above 78 years at the point of depression diagnosis (the mean age of the patients who were diagnosed with dementia during follow-up) translated to receiving a dementia diagnosis 9 months earlier than average. Having a diagnosis of recurrent depressive disorder or a previous diagnosis of depression were associated with a dementia diagnosis almost 14 months later than average, and treatment with CBT to 11 months later. Presenting with cognitive problems recorded on the HoNOS65+ instrument translated to a dementia diagnosis almost 17 months sooner than average. Table 3 shows time to dementia diagnosis according to severity of cognitive problems rated by clinicians on the HoNOS65+ subscale. Those who were scored to have no or minor cognitive problems at the time of their depression diagnosis progressed to a diagnosis of dementia in 3-4 years, and those with moderate to severe problems in under 2 years.

For peer review only

Table 3: Mean time from depression diagnosis to dementia diagnosis per score increase on the HoNOS65+ cognitive problems subscale recorded at the time of depression diagnosis, in 806 patients who were subsequently diagnosed with dementia.

HoNOS65+ cognitive problems scores	Mean time from depression diagnosis to dementia diagnosis in years (95% CI)
0 = no problem	3.77 (3.44 - 4.11)
1 = minor problem requiring no action	2.94 (2.67 - 3.20)
2 = mild problem but definitely present	2.05 (1.75 - 2.35)
3 = moderately severe problem	1.81 (1.39 - 2.22)
4 = severe to very severe problem	1.36 (0.25 - 2.46)

For peer review only

Discussion

This study showed in a cohort of over 3,500 older adults seen in secondary mental health services for depression that presenting at an older age, with new-onset depression and more severe cognitive problems, and not being treated with CBT, were each predictive of being subsequently diagnosed with dementia.

There is ongoing debate as to whether recurrent depression [26, 27], or history of depression [28] carry an increased risk of dementia. We found that a diagnosis of recurrent depressive disorder was protective in relation to overall dementia risk, and in those who went on to develop dementia, recurrent depression was associated with a slower progression to a diagnosis which translated to over a year later than the average time. We postulate that the 187 patients with recurrent depression who developed dementia (23.2% of the total 806 patients who developed dementia) were more likely to be suffering initially from mood disorder distinct from incipient dementia, and so development of dementia overall came later. The remaining patients who were categorised as having new-onset depression may have been following a trajectory of newly emergent, steadily increasing depressive symptoms which have previously been shown to characterise prodromal dementia [7-9]; and reached a threshold of severity, at which point they reached secondary mental health services and the index diagnosis of depression was made. Methodological differences complicate comparisons between findings relating to the relationship between recurrent depression and dementia risk, as studies vary in their classifications of new-onset and recurrent depression, the time frame, and whether it was self-reported or clinically diagnosed. In this study, the severity and persistence of clinical symptoms across the cohort warranted a referral to secondary mental health services. The results are consistent with the findings from another large, community-based cohort study, in showing that a recent episode of depression in later life is a strong predictor of progression to dementia [29].

It is not surprising that more severe cognitive impairment in patients with late-life depression was associated with a higher dementia risk and predicted that a diagnosis of dementia would be made sooner. Past studies have shown that recently active depression in combination with MCI confers particularly high risk for Alzheimer's

disease; 40% progressed to Alzheimer's disease within an average follow-up time of just over 2 years [29]. Similarly, in a cohort of older adults with high depressive symptoms in German primary care services, co-existence of marked cognitive deficits was a distinguishing prognostic marker of subsequent dementia within the 6-year follow-up period [30]. Against this background, the authors discouraged the attribution of cognitive impairment in late-life depression to "depressive pseudodementia" as it can be a strong indication of incipient dementia that warrants further investigation; a view supported by our study. A case-control sample from the PAQUID cohort demonstrated a similar emergence of depressive symptoms and cognitive and functional decline in the years preceding the dementia phase of AD [31]. In that cohort, progression of cognitive decline was accompanied by depressive symptoms, and as a consequence of continued worsening, independence in activities of daily living then began to deteriorate.

Regarding the potential effects of antidepressant medication, although not significant after accounting for multiple comparisons, those prescribed 'any antidepressant' appeared to be at a higher risk of developing dementia in our cohort. This is in line with the growing evidence for this association from observational studies [32], and is consistent with a large, matched, prospective cohort study that found that antidepressant monotherapy was associated with an over two-fold increased dementia risk in those aged over 60 after adjusting for multiple confounders, including depression [33]. Poor antidepressant response has been reported to be indicative of an especially increased risk of dementia in patients with cognitive impairment [34], and it's likely that a number of patients in our study were referred to secondary care following treatment failure because over half had been prescribed antidepressants prior to reaching the service. However, this is challenging to disentangle if the efficacy of antidepressants might generally be reduced in patients aged over 65, as suggested by a number of meta-analyses [35, 36]. As over 85% of our cohort were prescribed antidepressant drugs following their depression diagnosis, the analysis lacked the comparison group required to elucidate whether this indeed had an influence on dementia risk. Also, as only a crude measure of depression severity was adjusted for, it's possible that the few patients who were not prescribed an antidepressant might have suffered from milder forms of depression, meaning that the association with dementia risk reflects differences in depression symptomology rather than

antidepressant treatment. Potentially of note is that the ‘any antidepressant’ group yielded an increased dementia risk, but the SSRI group did not. This could be due to a lack of statistical power as the number of patients prescribed specifically SSRIs was lower, or it could indicate that the inclusion of antidepressants with a higher anticholinergic burden in this group (e.g. tricyclic antidepressants) might have contributed to the increased hazard of developing dementia [37]. Robust analyses of the efficacy of specific pharmacotherapies are often outside the scope of observational cohort studies, and from our study we cannot infer any neuroprotective effects of antidepressants. Controlled trials are yet to identify any candidate antidepressant treatments that can improve cognitive outcomes in older adults with depression [38, 39], except for some small short-term improvements in executive functioning [40, 41].

After adjusting for multiple confounders, undergoing CBT was associated with an almost 30% reduced risk of later being diagnosed with dementia. One likely explanation is that the patients who were offered CBT systematically differed from those not offered the intervention beyond what was possible to control for through adjusting for confounders, particularly in relation to their severity of depressive and cognitive symptoms. On the other hand, cognitive behavioural techniques are known to reduce some of the typical risk factors for dementia such as lack of cognitive, physical or social activity, a restricted diet or sleep disorders [42, 43]. Further, CBT for insomnia has shown benefits to executive functioning in a small randomized study of people with MCI and insomnia [44]. Whether CBT needs to be adapted for late-life depression and could show benefits to cognitive decline requires further exploration [45].

Of note, although not significant after adjustment for multiple comparisons, the presence of non-accidental self-injury and psychotic symptoms appeared to be associated with a lower dementia risk. These results are consistent with a previous study of late-life depression that utilised this data source, in which certain depressive symptoms, i.e. guilt feelings, impaired concentration, disturbed sleep, and delusions, were associated with a lower mortality risk in people with late-life depression [46]. The study also showed that severity of problems related to depressed mood on the HoNOS65+ subscale predicted mortality only when patients with new-onset depression were considered [46]. This could be related to the sample being drawn

For peer review only

from a secondary care provider for which a certain severity threshold needs to be met, and might explain why depression severity according to the HoNOS65+ subscale was not associated with an increased risk of dementia in the present study.

Strengths and limitations

Strengths of this study include the large, population-based sample in routine clinical care. The main limitations relate to the generalisability of the findings drawn from this selective cohort. To be captured in the study patients needed a secondary care diagnosis of depression, meaning these findings might not generalise to patients with milder depressive symptoms treated in primary care or those not readily seeking a depression diagnosis [47]. Crucially, the findings from examining the duration between depression and dementia diagnosis relate to the time taken to receive a diagnosis of dementia in SLaM services, rather than the time taken to develop the diagnosable symptoms of dementia. This duration is influenced by potential barriers to timely diagnosis; service, provider, patient and care-giver factors contribute to delayed dementia diagnosis [48]. A review of the literature on missed and delayed diagnosis of dementia found symptom severity and degree of impairment are important predictors of diagnostic sensitivity [48]. Additionally, late-life depression confers an increased mortality risk, both in relation to suicide and when suicidality is accounted for [49], and the sub-cohort of patients who received both depression and dementia diagnoses in SLaM was selective to the 806 patients who survived long enough to receive a dementia diagnosis.

Of the 806 patients diagnosed with dementia, we found that 25% received an initial diagnosis of unspecified dementia and only 44% a diagnosis of Alzheimer's disease. This is lower than the expected incidence of Alzheimer's disease in two-thirds of dementia cases [50] and likely to reflect the naturalistic nature of the data, whereby the specific subtype is often recorded as unspecified until further investigations are conducted or more specific symptoms emerge. Subsequently the recording of specific subtypes increases if the whole record is considered [51, 52].

Medication prescription was ascertained either within the 2 years before or after the index date of first depression diagnosis through natural language processing, but the time-dependent nature of prescribing could not be accounted for. Further, the output

For peer review only

of natural language processing depends on the accuracy and quality of data entry, which varies by individual clinician and is compromised through the use of jargon, idiosyncratic abbreviations or misspellings [17]. Although it has been shown that precision and recall are relatively high for the medication natural language processing application [17], there remains a risk under- or overestimating the true prescribing prevalence, especially as adherence to medication cannot be established [53]. Lastly, although we incorporated a wide range of potential confounders in our analysis, causal inferences cannot be readily be drawn in an observational study of this nature.

Conclusions

The aim of our study was to highlight clinical markers of increased risk and accelerated progression to dementia in late-life depression. A knowledge of the indicators of the preclinical phase of dementia may be crucial to aid advancement in the identification, and consequently the management, of the disease at the earliest possible stage. As patients at the earliest stage of dementia are most likely to benefit from intervention to delay progression, future efforts to improve the detection of dementia should focus on recognizing subtle, early manifestations of disease. By examining the temporal relationships between characteristics within late-life depression and progression to dementia, in the context of South London secondary mental health service users, we have highlighted features that may indicate higher risk of and sooner progression to dementia. In older patients presenting with new-onset depression and/or more problematic cognitive symptoms, clinicians should suspect a diagnosis of dementia. It may be beneficial to closely monitor these patients for signs of cognitive deterioration suggestive of dementia, to facilitate the earliest possible detection and diagnosis. These patients might also form an interesting group for which novel pharmacological or psychosocial interventions could be developed.

Author Contributions:

GeP, CM, RS and DA conceived the study. GeP, CM and GaP performed the analysis and interpreted the data. GeP and CM wrote the manuscript. NK, MS, DA, RS provided expertise and feedback. All authors critically revised the manuscript and approved the final version of the study.

For peer review only

Data Availability Statement:

Deidentified patient data was obtained via the Clinical Record Interactive Search system (CRIS) at the National Institute for Health Research (NIHR) Biomedical Research Centre (BRC) at South London and Maudsley NHS Foundation Trust and the Institute of Psychiatry, Psychology & Neuroscience at King's College London. The data accessed by CRIS remain within an NHS firewall and governance is provided by a patient-led oversight committee. There are no additional unpublished data available.

For peer review only

References:

1. Byers, A.L. and K. Yaffe, *Depression and risk of developing dementia.* Nat Rev Neurol, 2011. **7**(6): p. 323-31.
2. Heo, M., et al., *Population projection of US adults with lifetime experience of depressive disorder by age and sex from year 2005 to 2050.* Int J Geriatr Psychiatry, 2008. **23**(12): p. 1266-70.
3. Cherbuin, N., S. Kim, and K.J. Anstey, *Dementia risk estimates associated with measures of depression: a systematic review and meta-analysis.* BMJ Open, 2015. **5**(12): p. e008853.
4. Ownby, R.L., et al., *Depression and risk for Alzheimer disease: systematic review, meta-analysis, and metaregression analysis.* Arch Gen Psychiatry, 2006. **63**(5): p. 530-8.
5. Diniz, B.S., et al., *Late-life depression and risk of vascular dementia and Alzheimer's disease: systematic review and meta-analysis of community-based cohort studies.* Br J Psychiatry, 2013. **202**(5): p. 329-35.
6. Livingston, G., et al., *Dementia prevention, intervention, and care.* Lancet, 2017. **390**(10113): p. 2673-2734.
7. Kaup, A.R., et al., *Trajectories of Depressive Symptoms in Older Adults and Risk of Dementia.* JAMA Psychiatry, 2016. **73**(5): p. 525-31.
8. Mirza, S.S., et al., *10-year trajectories of depressive symptoms and risk of dementia: a population-based study.* Lancet Psychiatry, 2016. **3**(7): p. 628-35.
9. Singh-Manoux, A., et al., *Trajectories of Depressive Symptoms Before Diagnosis of Dementia: A 28-Year Follow-up Study.* JAMA Psychiatry, 2017. **74**(7): p. 712-718.
10. Almeida, O.P., et al., *Depression as a modifiable factor to decrease the risk of dementia.* Transl Psychiatry, 2017. **7**(5): p. e1117.
11. Mirza, S.S., et al., *Depressive symptoms predict incident dementia during short- but not long-term follow-up period.* Alzheimers Dement, 2014. **10**(5 Suppl): p. S323-S329.e1.
12. Ismail, Z., et al., *Neuropsychiatric symptoms as early manifestations of emergent dementia: Provisional diagnostic criteria for mild behavioral impairment.* Alzheimers Dement, 2016. **12**(2): p. 195-202.
13. Jorm, A.F., *History of depression as a risk factor for dementia: an updated review.* Aust N Z J Psychiatry, 2001. **35**(6): p. 776-81.
14. Donovan, N.J., et al., *Longitudinal Association of Amyloid Beta and Anxious-Depressive Symptoms in Cognitively Normal Older Adults.* Am J Psychiatry, 2018. **175**(6): p. 530-537.
15. Panza, F., et al., *Late-life depression, mild cognitive impairment, and dementia: possible continuum?* Am J Geriatr Psychiatry, 2010. **18**(2): p. 98-116.
16. Fernandes, A.C., et al., *Development and evaluation of a de-identification procedure for a case register sourced from mental health electronic records.* BMC Med Inform Decis Mak, 2013. **13**: p. 71.
17. Perera, G., et al., *Cohort profile of the South London and Maudsley NHS Foundation Trust Biomedical Research Centre (SLaM BRC) Case Register: current status and recent enhancement of an Electronic Mental Health Record-derived data resource.* BMJ Open, 2016. **6**(3): p. e008721.

For peer review only

18. Cunningham, H., et al., *Getting more out of biomedical documents with GATE's full lifecycle open source text analytics.* PLoS computational biology, 2013. **9**(2): p. e1002854.
19. World Health, O., *International statistical classification of diseases and related health problems*. Vol. 1. 2004: World Health Organization.
20. Uk, G.O.V., *English indices of deprivation 2010*. 2016, Accessed.
21. Burns, A., et al., *Health of the Nation Outcome Scales for elderly people (HoNOS 65+).* Br J Psychiatry, 1999. **174**: p. 424-7.
22. StataCorp, L.P., *Stata: release 13-statistical software.* College Station, TX, 2013.
23. Oudshoorn, C.G.M., S. Buuren, and J.L.A. Rijckevorsel, *Flexible multiple imputation by chained equations of the AVO-95 survey*. 1999: TNO Prevention and Health Leiden.
24. Rubin, D.B., *Multiple imputation for nonresponse in surveys*. Vol. 81. 2004: John Wiley & Sons.
25. Stewart, R., et al., *The South London and Maudsley NHS Foundation Trust Biomedical Research Centre (SLAM BRC) case register: development and descriptive data.* BMC Psychiatry, 2009. **9**: p. 51.
26. Dotson, V.M., M.A. Beydoun, and A.B. Zonderman, *Recurrent depressive symptoms and the incidence of dementia and mild cognitive impairment.* Neurology, 2010. **75**(1): p. 27-34.
27. Barnes, D.E., et al., *Midlife vs late-life depressive symptoms and risk of dementia: differential effects for Alzheimer disease and vascular dementia.* Arch Gen Psychiatry, 2012. **69**(5): p. 493-8.
28. Wetherell, J.L., et al., *History of depression and other psychiatric illness as risk factors for Alzheimer disease in a twin sample.* Alzheimer Dis Assoc Disord, 1999. **13**(1): p. 47-52.
29. Gallagher, D., et al., *Depression and Risk of Alzheimer Dementia: A Longitudinal Analysis to Determine Predictors of Increased Risk among Older Adults with Depression.* Am J Geriatr Psychiatry, 2018. **26**(8): p. 819-827.
30. Heser, K., et al., *Late-Life Depressive Symptoms and Lifetime History of Major Depression: Cognitive Deficits are Largely Due to Incipient Dementia rather than Depression.* J Alzheimers Dis, 2016. **54**(1): p. 185-99.
31. Amieva, H., et al., *Prodromal Alzheimer's disease: successive emergence of the clinical symptoms.* Ann Neurol, 2008. **64**(5): p. 492-8.
32. Wang, Y.C., et al., *Increased Risk of Dementia in Patients with Antidepressants: A Meta-Analysis of Observational Studies.* Behav Neurol, 2018. **2018**: p. 5315098.
33. Kodesh, A., et al., *Exposure to Antidepressant Medication and the Risk of Incident Dementia.* Am J Geriatr Psychiatry, 2019. **27**(11): p. 1177-1188.
34. Modrego, P.J. and J. Ferrández, *Depression in patients with mild cognitive impairment increases the risk of developing dementia of Alzheimer type: a prospective cohort study.* Arch Neurol, 2004. **61**(8): p. 1290-3.
35. Tedeschini, E., et al., *Efficacy of antidepressants for late-life depression: a meta-analysis and meta-regression of placebo-controlled randomized trials.* J Clin Psychiatry, 2011. **72**(12): p. 1660-8.
36. Tham, A., et al., *Efficacy and tolerability of antidepressants in people aged 65 years or older with major depressive disorder - A systematic review and a meta-analysis.* J Affect Disord, 2016. **205**: p. 1-12.

37. Bishara, D., et al., *Anticholinergic effect on cognition (AEC) of drugs commonly used in older people.* Int J Geriatr Psychiatry, 2017. **32**(6): p. 650-656.
38. Nebes, R.D., et al., *Persistence of cognitive impairment in geriatric patients following antidepressant treatment: a randomized, double-blind clinical trial with nortriptyline and paroxetine.* J Psychiatr Res, 2003. **37**(2): p. 99-108.
39. Bhalla, R.K., et al., *Persistence of neuropsychologic deficits in the remitted state of late-life depression.* Am J Geriatr Psychiatry, 2006. **14**(5): p. 419-27.
40. Butters, M.A., et al., *Changes in cognitive functioning following treatment of late-life depression.* Am J Psychiatry, 2000. **157**(12): p. 1949-54.
41. Devanand, D.P., et al., *Sertraline treatment of elderly patients with depression and cognitive impairment.* Int J Geriatr Psychiatry, 2003. **18**(2): p. 123-30.
42. Reid, L.D., F.E. Avens, and A.A. Walf, *Cognitive behavioral therapy (CBT) for preventing Alzheimer's disease.* Behav Brain Res, 2017. **334**: p. 163-177.
43. Sommerlad, A., et al., *Association of social contact with dementia and cognition: 28-year follow-up of the Whitehall II cohort study.* PLoS Med, 2019. **16**(8): p. e1002862.
44. Cassidy-Eagle, E., et al., *Neuropsychological Functioning in Older Adults with Mild Cognitive Impairment and Insomnia Randomized to CBT-I or Control Group.* Clin Gerontol, 2018. **41**(2): p. 136-144.
45. Dafsari, F.S., et al., *Cognitive behavioural therapy for the treatment of late life depression: study protocol of a multicentre, randomized, observer-blinded, controlled trial (CBTlate).* BMC Psychiatry, 2019. **19**(1): p. 423.
46. Cai, W., et al., *Predictors of mortality in people with late-life depression: A retrospective cohort study.* Journal of Affective Disorders, 2020.
47. Winblad, B., et al., *Defeating Alzheimer's disease and other dementias: a priority for European science and society.* Lancet Neurol, 2016. **15**(5): p. 455-532.
48. Bradford, A., et al., *Missed and delayed diagnosis of dementia in primary care: prevalence and contributing factors.* Alzheimer Dis Assoc Disord, 2009. **23**(4): p. 306-14.
49. Hall, C.A. and C.F. Reynolds-Iii, *Late-life depression in the primary care setting: challenges, collaborative care, and prevention.* Maturitas, 2014. **79**(2): p. 147-52.
50. Corriveau, R.A., et al., *Alzheimer's Disease-Related Dementias Summit 2016: National research priorities.* Neurology, 2017. **89**(23): p. 2381-2391.
51. Mueller, C., et al., *Hospitalization in people with dementia with Lewy bodies: Frequency, duration, and cost implications.* Alzheimer's & Dementia: Diagnosis, Assessment & Disease Monitoring, 2018. **10**: p. 143-152.
52. Mueller, C., et al., *Polypharmacy in people with dementia: Associations with adverse health outcomes.* Experimental Gerontology, 2018.
53. Cai, W., et al., *Predictors of cerebrovascular event reoccurrence in patients with depression: a retrospective cohort study.* BMJ Open, 2020. **10**(1): p. e031927.

For peer review only

Figure legends/captions:

Figure 1
Title: Flowchart showing cohort generation and outcomes.

Figure 2
Title: Plot of hazard ratios for risk of dementia diagnosis in a multivariate Cox regression model adjusted for all covariates in 3,659 patients with late-life depression, of whom 806 developed dementia.
Legend:
Coloured points indicate ***p*<.002 (Bonferroni corrected)
[a] at or closest to the time of depression diagnosis.
[b] per 10 unit increase in IMD score.
[c] Recurrent depressive disorder diagnosis or previous diagnosis of depression.
[d] Health of the Nation Outcome Scale 65+ (HoNOS65+) subscale scores 0-4 (0 = least severe, 4 = most severe status). Values represent frequencies of patients scored as experiencing problems (score 2-4) in that domain.
[e] time-variable-interactions describing how the hazard in the variable reported above changes per year.

Figure 3
Title: Impact of significant risk factors on time to dementia diagnosis in 806 patients with late-life depression who were subsequently diagnosed with dementia during follow-up.
Legend:
[a] at or closest to the time of depression diagnosis.
[b] Health of the Nation Outcome Scale 65+ (HoNOS65+) subscale scores 0-4 (0 = least severe, 4 = most severe status). Values represent frequencies of patients scored as experiencing problems (score 2-4) in that domain.
[c] Recurrent depressive disorder diagnosis or previous diagnosis of depression.

For peer review only

Figure 1. Flowchart showing cohort generation and outcomes.

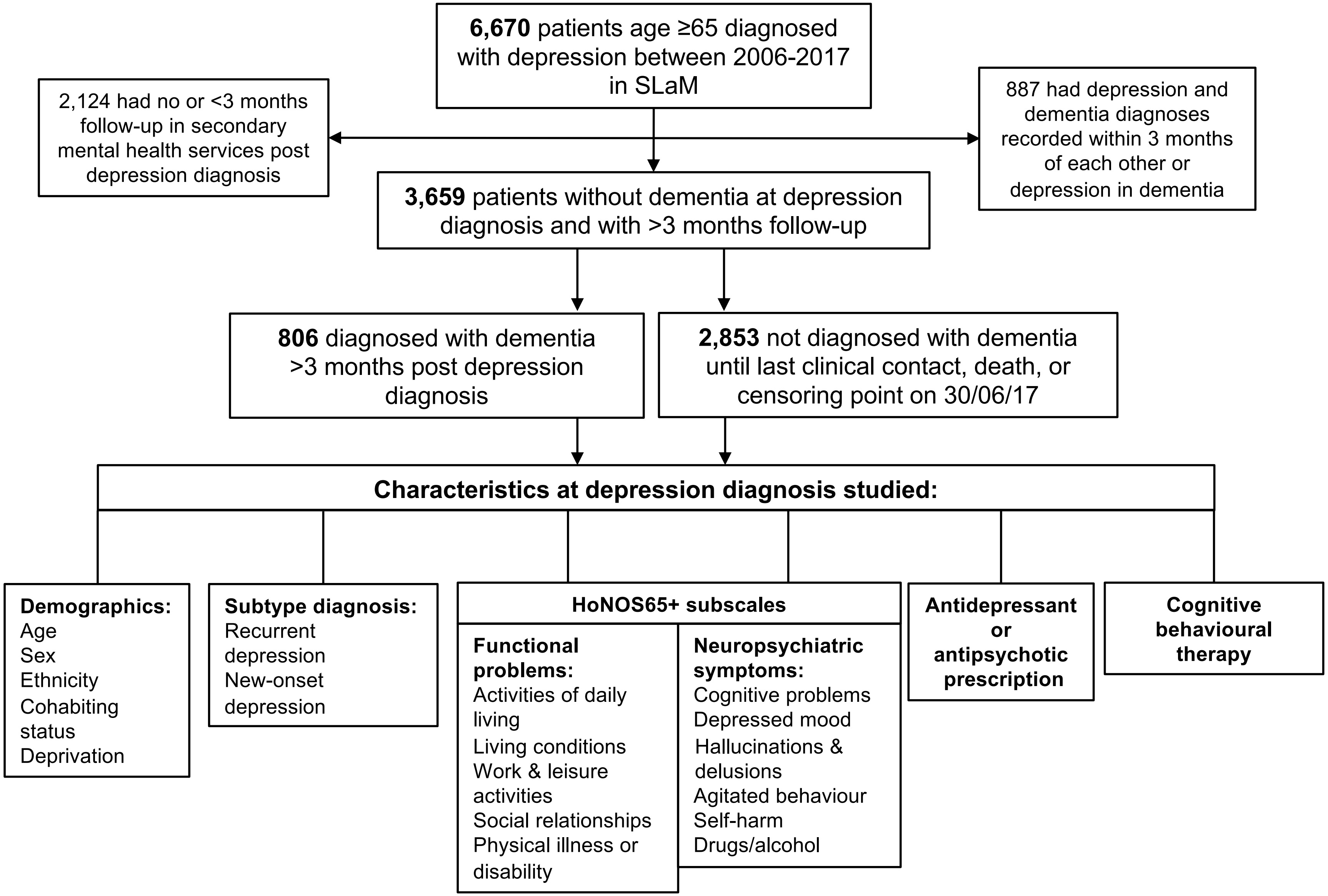

Figure 2. Plot of hazard ratios for risk of dementia diagnosis in a multivariate Cox regression model adjusted for all covariates in 3,659 patients with late-life depression, of whom 806 developed dementia.

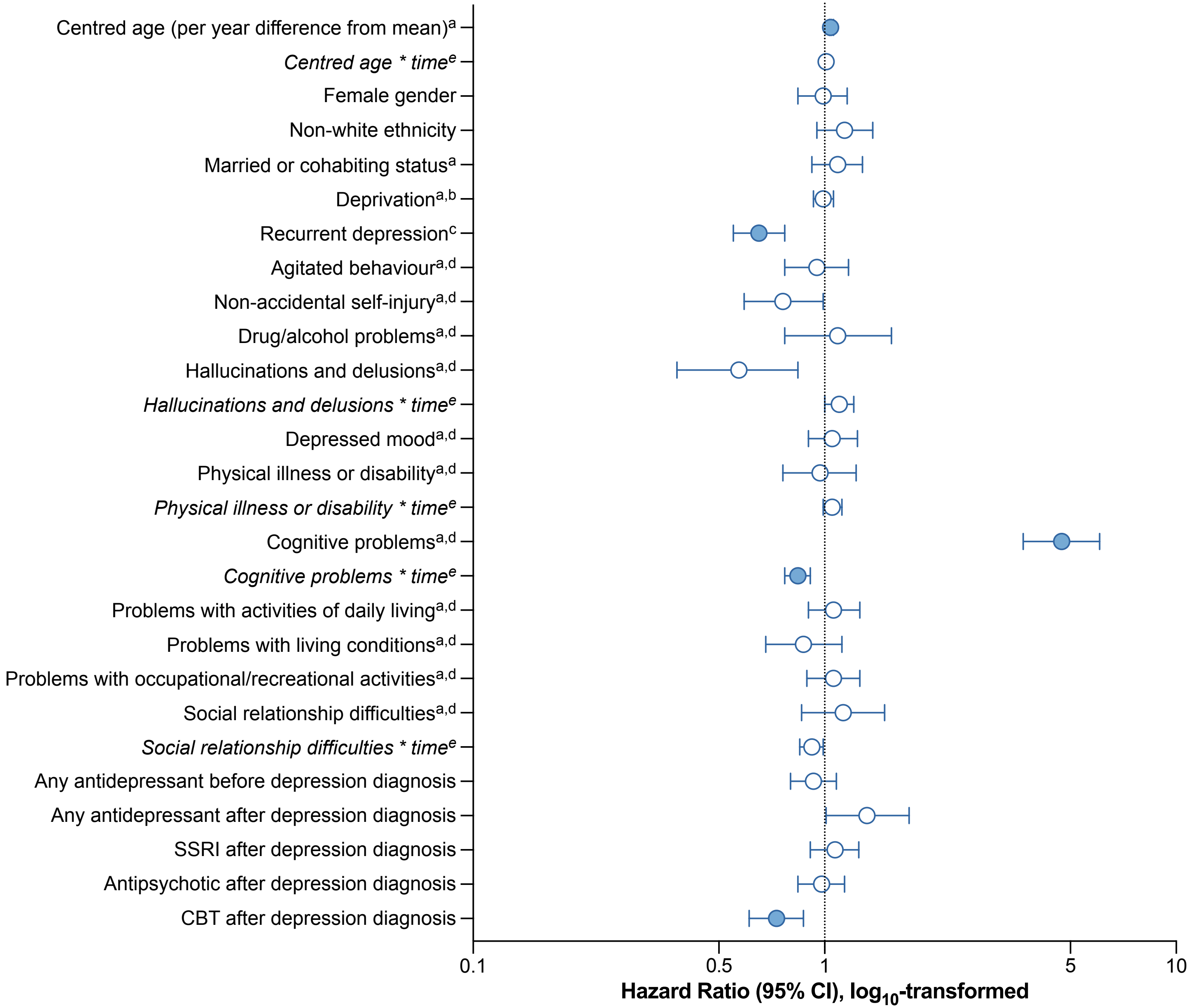

Figure 3. Impact of significant risk factors on time to dementia diagnosis in 806 patients with late-life depression who were subsequently diagnosed with dementia during follow-up.

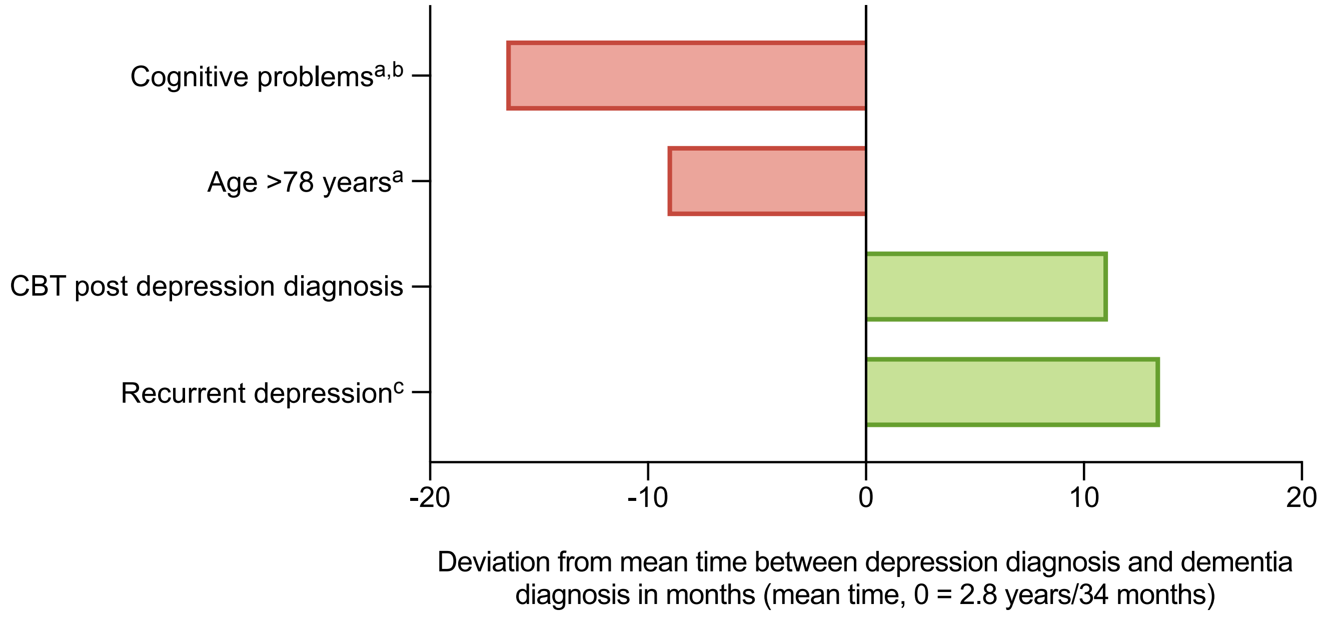

Supplementary Table 1: Multivariate Cox proportional hazards models analysing associations between predictor variables and risk of dementia diagnosis (adjusted hazard ratio (95% CI)) in the complete case sample of 2,952 late-life depression patients, of whom 717 (24.3%) were diagnosed with dementia during the study follow-up.

**Predictor variables**	**Model 1** Adjusted for age, gender, deprivation	**Model 2** Model 1 + depressed mood[b] + cognitive problems[b]	**Model 3** Adjusted for all predictor variables
**Demographics[a]**			
Centred age (per year difference from mean)	1.07 (1.06-1.08)**	1.05 (1.03-1.06)**	1.04 (1.03-1.06)**
*Centred age * time[c]*		*1.01 (1.00-1.01)**	*1.01 (1.00-1.01)**
Female gender	0.88 (0.75-1.03)	0.87 (0.75-1.03)	0.90 (0.76-1.06)
Non-white ethnicity	1.33 (1.10-1.61)*	1.21 (1.00-1.46)*	1.14 (0.94-1.39)
Married or cohabiting status[a]	1.04 (0.88-1.23)	1.08 (0.91-1.27)	1.07 (0.90-1.27)
Deprivation (per 10 unit increase in IMD score)	1.01 (0.95-1.08)	0.99 (0.93-1.06)	1.00 (0.94-1.06)
Recurrent depressive disorder diagnosis or previous diagnosis of depression	0.53 (0.39-0.70)**	0.71 (0.60-0.84)**	0.72 (0.60-0.86)**
*Recurrent depression * time[c]*	*1.08 (1.01-1.15)**		
**HoNOS65+ mental and physical health problems[a,b]**			
Agitated behaviour	1.06 (0.87-1.30)	0.88 (0.72-1.08)	0.97 (0.78-1.21)
Non-accidental self-injury	0.70 (0.54-0.90)*	0.69 (0.53-0.91)*	0.77 (0.59-1.01)
Drug/alcohol problems	1.04 (0.72-1.50)	0.98 (0.68-1.41)	1.08 (0.76-1.53)
Hallucinations and delusions	0.84 (0.67-1.07)	0.53 (0.36-0.78)**	0.52 (0.35-0.78)*
*Hallucinations and delusion * time[c]*		*1.10 (1.00-1.21)**	*1.13 (1.02-1.25)**
Depressed mood	1.09 (0.94-1.27)	1.00 (0.86-1.16)	1.07 (0.90-1.26)
Physical illness or disability	1.27 (1.08-1.48)*	1.14 (0.98-1.34)	0.90 (0.70-1.16)
*Physical illness or disability * time[c]*			*1.06 (0.99-1.13)*
Cognitive problems	4.84 (3.75-6.24)**	4.91 (3.80-6.34)**	4.66 (3.58-6.08)**
*Cognitive problems * time[c]*	*0.83 (0.77-0.90)***	*0.83 (0.77-0.90)***	*0.83 (0.76-0.90)***
**HoNOS65+ functional problems[a,b]**			
Activities of daily living	1.38 (1.18-1.60)**	1.10 (0.93-1.29)	1.04 (0.87-1.24)
Living conditions	1.05 (0.83-1.31)	0.90 (0.71-1.15)	0.90 (0.71-1.16)
Occupational/recreational activities	1.11 (0.95-1.29)	1.02 (0.86-1.20)	1.06 (0.89-1.27)
Social relationships	1.09 (0.83-1.42)	1.04 (0.79-1.36)	1.14 (0.86-1.51)
*Social relationships * time[c]*	*0.92 (0.85-0.99)**	*0.92 (0.86-1.00)**	*0.91 (0.84-0.98)**
**Psychotropic use: in 2 years pre-depression diagnosis**			
Antidepressant	0.86 (0.74-1.00)*	0.88 (0.76-1.03)	0.91 (0.78-1.06)
**Psychotropic use: post-depression diagnosis**			
Any antidepressant	1.12 (0.86-1.46)	1.17 (0.90-1.52)	1.30 (0.97-1.73)
SSRI	1.03 (0.88-1.19)	1.00 (0.86-1.17)	0.98 (0.83-1.15)
Antipsychotic	0.86 (0.74-1.00)*	0.85 (0.73-0.99)*	0.98 (0.83-1.16)
**Cognitive behavioural therapy**			
Received CBT post-depression diagnosis	0.59 (0.50-0.70)**	0.66 (0.55-0.79)**	0.70 (0.58-0.85)**

*$p<.05$
**$p<.002$ (Bonferroni corrected)
[a] at or closest to the time of depression diagnosis
[b] Health of the Nation Outcome Scale 65+ (HoNOS65+) subscale scores 0-4 (0 = least severe, 4 = most severe status). Values represent frequencies of patients scored as experiencing problems (score 2-4) in that domain.
[c] time-variable-interactions describing how the hazard in the variable reported above changes per year.

For peer review only

STROBE Statement—Checklist of items that should be included in reports of ***cohort studies***

Page and line numbers refer to those in the PDF proof of the manuscript entitled "Clinical factors associated with progression to dementia in people with late-life depression: a cohort study of patients in secondary care".

	Item No	Recommendation	Page No
**Title and abstract**	1	(*a*) Indicate the study's design with a commonly used term in the title or the abstract	Page 6
		(*b*) Provide in the abstract an informative and balanced summary of what was done and what was found	Page 8
**Introduction**			
Background/rationale	2	Explain the scientific background and rationale for the investigation being reported	Page 10-11
Objectives	3	State specific objectives, including any prespecified hypotheses	Page 11, lines 5-14
**Methods**			
Study design	4	Present key elements of study design early in the paper	Page 11, lines 9-14
Setting	5	Describe the setting, locations, and relevant dates, including periods of recruitment, exposure, follow-up, and data collection	Page 12, lines 38-59
Participants	6	(*a*) Give the eligibility criteria, and the sources and methods of selection of participants. Describe methods of follow-up	Page 12, lines 38-59<br>Figure 1
		(*b*) For matched studies, give matching criteria and number of exposed and unexposed	
Variables	7	Clearly define all outcomes, exposures, predictors, potential confounders, and effect modifiers. Give diagnostic criteria, if applicable	Page 13
Data sources/ measurement	8*	For each variable of interest, give sources of data and details of methods of assessment (measurement). Describe comparability of assessment methods if there is more than one group	Page 12, lines 9-30<br>Page 13
Bias	9	Describe any efforts to address potential sources of bias	
Study size	10	Explain how the study size was arrived at	Page 16, lines 9-20
Quantitative variables	11	Explain how quantitative variables were handled in the analyses. If applicable, describe which groupings were chosen and why	Page 13
Statistical methods	12	(*a*) Describe all statistical methods, including those used to control for confounding	Page 14
		(*b*) Describe any methods used to examine subgroups and interactions	
		(*c*) Explain how missing data were addressed	
		(*d*) If applicable, explain how loss to follow-up was addressed	
		(*e*) Describe any sensitivity analyses	
**Results**			
Participants	13*	(a) Report numbers of individuals at each stage of study—eg numbers potentially eligible, examined for eligibility, confirmed eligible, included in the study, completing follow-up, and analysed	Page 16, lines 9-25<br>Figure 1
		(b) Give reasons for non-participation at each stage	
		(c) Consider use of a flow diagram	

For peer review only - http://bmjopen.bmj.com/site/about/guidelines.xhtml

Descriptive data	14*	(a) Give characteristics of study participants (eg demographic, clinical, social) and information on exposures and potential confounders<br>(b) Indicate number of participants with missing data for each variable of interest<br>(c) Summarise follow-up time (eg, average and total amount)	Page 16<br>Table 1
Outcome data	15*	Report numbers of outcome events or summary measures over time	Page 16<br>Table 1

For peer review only

For peer review only - http://bmjopen.bmj.com/site/about/guidelines.xhtml

Main results	16	(*a*) Give unadjusted estimates and, if applicable, confounder-adjusted estimates and their precision (eg, 95% confidence interval). Make clear which confounders were adjusted for and why they were included<br>(*b*) Report category boundaries when continuous variables were categorized<br>(*c*) If relevant, consider translating estimates of relative risk into absolute risk for a meaningful time period	Page 18<br>Table 2
Other analyses	17	Report other analyses done—eg analyses of subgroups and interactions, and sensitivity analyses	Page 18
**Discussion**			
Key results	18	Summarise key results with reference to study objectives	Page 22, lines 5-11
Limitations	19	Discuss limitations of the study, taking into account sources of potential bias or imprecision. Discuss both direction and magnitude of any potential bias	Page 25 line 22 – page 26 line 31
Interpretation	20	Give a cautious overall interpretation of results considering objectives, limitations, multiplicity of analyses, results from similar studies, and other relevant evidence	Page 22-24
Generalisability	21	Discuss the generalisability (external validity) of the study results	Page 25 lines 22-31
**Other information**			
Funding	22	Give the source of funding and the role of the funders for the present study and, if applicable, for the original study on which the present article is based	Page 6, lines 47-58

*Give information separately for exposed and unexposed groups.

Note: An Explanation and Elaboration article discusses each checklist item and gives methodological background and published examples of transparent reporting. The STROBE checklist is best used in conjunction with this article (freely available on the Web sites of PLoS Medicine at http://www.plosmedicine.org/, Annals of Internal Medicine at http://www.annals.org/, and Epidemiology at http://www.epidem.com/). Information on the STROBE Initiative is available at http://www.strobe-statement.org.

For peer review only
